# Temperature‐Dependent Phase Transition in WS_2_ for Reinforcing Band‐to‐Band Tunneling and Photoreactive Random Access Memory Application

**DOI:** 10.1002/smsc.202300202

**Published:** 2023-11-21

**Authors:** Gunhoo Woo, Jinill Cho, Heejung Yeom, Min Young Yoon, Geon Woong Eom, Muyoung Kim, Jihun Mun, Hyo Chang Lee, Hyeong-U Kim, Hocheon Yoo, Taesung Kim

**Affiliations:** ^1^ SKKU Advanced Institute of Nanotechnology (SAINT) Sungkyunkwan University (SKKU) Suwon 16419 Republic of Korea; ^2^ Department of Mechanical Engineering Sungkyunkwan University (SKKU) Suwon 16419 Republic of Korea; ^3^ Advanced Instrumentation Institute Korea Research Institute of Standards and Science (KRISS) Daejeon 34113 Republic of Korea; ^4^ Department of Physics Chungnam National University (CNU) Daejeon 35018 Republic of Korea; ^5^ Department of Plasma Engineering Korea Institute of Machinery and Materials (KIMM) Daejeon 34103 Republic of Korea; ^6^ Department of Semiconductor Science Engineering and Technology Korea Aerospace University Goyang 10540 Republic of Korea; ^7^ School of Electronics and Information Engineering Korea Aerospace University Goyang 10540 Republic of Korea; ^8^ Department of Electronic Engineering Gachon University 1342 Seongnam-daero Seongnam 13120 Republic of Korea

**Keywords:** negative differential resistances, optoelectrical devices, phase modulations, plasma-enhanced chemical vapor depositions, random-access memories

## Abstract

In the era of big data, negative differential resistance (NDR) devices have attracted significant attention as a means of handling massive amounts of information. While 2D materials have been used to achieve NDR behavior, their intrinsic material characteristics have produced limited performance improvements. In this article, a facile phase modification method is presented via a plasma‐assisted sulfidation process to synthesize multiphased WS_2_ thin films, including distorted 1 T (D‐1 T) phase and 2 H phases for photoreactive NDR devices with *p*‐Si. The D‐1 T phase offers a feasible route to achieve high‐performance NDR devices with excellent stability and semimetallic properties. A comprehensive investigation of experimental and computational analyses elucidates the phase transition mechanism with various temperatures and electrical properties of D‐1 T WS_2_. In addition, optimizing electron tunneling in the multiple‐phased tungsten disulfide (MP‐WS_2_)/*p*‐Si heterojunction at MP‐WS_2_ with 77.4% D‐1 T phase results in superior NDR performance with a peak‐to‐valley current ratio of 13.8 and reliable photoreactive random‐access memory. This unique phase engineering process via plasma‐assisted sulfidation provides a pioneering perspective in functionalization and reliability for next‐generation nanoelectronics.

## Graphical abstract

The plasma‐assisted chemical‐vapor‐deposition‐based metal‐layer sulfidation method is used to synthesize phase‐tunable WS_2_ film according to regulating process temperature and achieve a large peak‐to‐valley current ratio value of 13.8 with a high light current of 0.21 μA under illumination state via multiple‐phased tungsten disulfide/*p*‐Si structure. The proposed functional device is applied as a photoreactive random‐access memory.

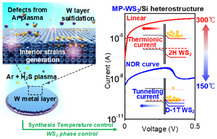

## 1 Introduction

The increasing demand for rapid computing speed and higher power efficiency to handle massive data in advanced computing technologies, such as artificial intelligence and internet‐of‐things,^[^ ^1^, ^2^, ^3^ ^]^ has spurred interest in negative differential resistance (NDR) devices. This device has been highlighted for its potential applications in multilevel logic,^[^ ^4^, ^5^, ^6^ ^]^ random‐access memory (RAM),^[^ ^7^, ^8^, ^9^ ^]^ and oscillator systems.^[^ ^10^, ^11^, ^12^ ^]^ The NDR phenomenon exhibits a drastic current drop at a specific voltage range and an N‐shaped current–voltage (*I–V*) curve under an applied bias. This unique current behavior provides several advantages in multiple threshold point^[^ ^5^, ^13^ ^]^ and resistive bistability.^[^ ^8^, ^14^ ^]^ According to the Wentzel–Kramers–Brillouin approximation, the performance of an NDR device is associated directly with the tunneling probability at the heterointerface aligned with the broken band structure.^[^ ^15^ ^]^ Therefore, the formation of a sharp contact (with a small thickness of tunneling barrier) and a small effective mass/bandgap of materials are crucial for prompt carrier tunneling transportation through a tunneling window.^[^ ^16^, ^17^ ^]^

Various band alignments (types I, II, and III) and the formation of van‐der‐Waals‐based sharp contacts with other semiconductors have been used to apply NDR devices using 2D materials.^[^ ^17^, ^18^, ^19^ ^]^ In contrast, despite the excellent availability of 2D materials, there are limitations in improving tunneling efficiency using only their intrinsic properties. Additional challenges, such as hexagonal boron nitride encapsulation and doping processes, have been explored to prevent degradation from external environments and improve the electron tunneling performance.^[^ ^20^, ^21^, ^22^ ^]^ Unfortunately, these processes are not feasible because of the increased manufacturing costs, complex fabrication, and device stability concerns. Hence, practical modification methods should be developed to realize high‐performance NDR devices and overcome the limitations of relying solely on the intrinsic properties of 2D materials. In this regard, distorted octahedral (distorted 1 T, D‐1 T) transition‐metal dichalcogenide (TMDC) has attracted interest because of its semimetallic properties and excellent stability under an ambient environment.^[^ ^23^, ^24^, ^25^ ^]^ The 1 T’‐phased TMDC is a quasi‐metal with a bandgap of approximately 0.12 eV.^[^ ^26^ ^]^ Moreover, their outstanding chemical and structural stability allowed the 1 T’‐phase TMDC to be applied in various research fields.^[^ ^27^ ^]^ These electronic properties and the stability of 1 T’‐phase materials make them suitable for tunneling device applications. Despite these merits, utilizing them is still challenging because of the incomplete large‐area thin‐film synthesis and an inconvenient phase‐control process.

In this context, multiple‐phased tungsten disulfide (MP‐WS_2_) thin films containing D‐1 T and 2 H phases deposited by plasma‐enhanced chemical vapor deposition (PE‐CVD) were used for robust and high‐performance NDR devices. The properties of D‐1 T in MP‐WS_2_ are determined by the synthesis times and temperatures. Based on the electrical characteristics of the MP‐WS_2_/*p*‐Si device, the correlation between the D‐1 T/2 H‐phase ratio and the electron tunneling intensity was analyzed, and the NDR performance can be optimized. The photoreactive RAM operated according to the electrical and optical signals at the current sweeping mode was implemented to exhibit the practicality of the proposed NDR device. Hence, D‐1 T WS_2_ is compatible as a functional channel material in NDR devices. The NDR performance is controlled by modifying the D‐1 T/2 H‐phase ratio. This article reports the synthesis of multiphase 2D materials using a plasma‐assisted sulfidation method.

## 2 Overview of the Phase Modulation Process

**Figure 1:**
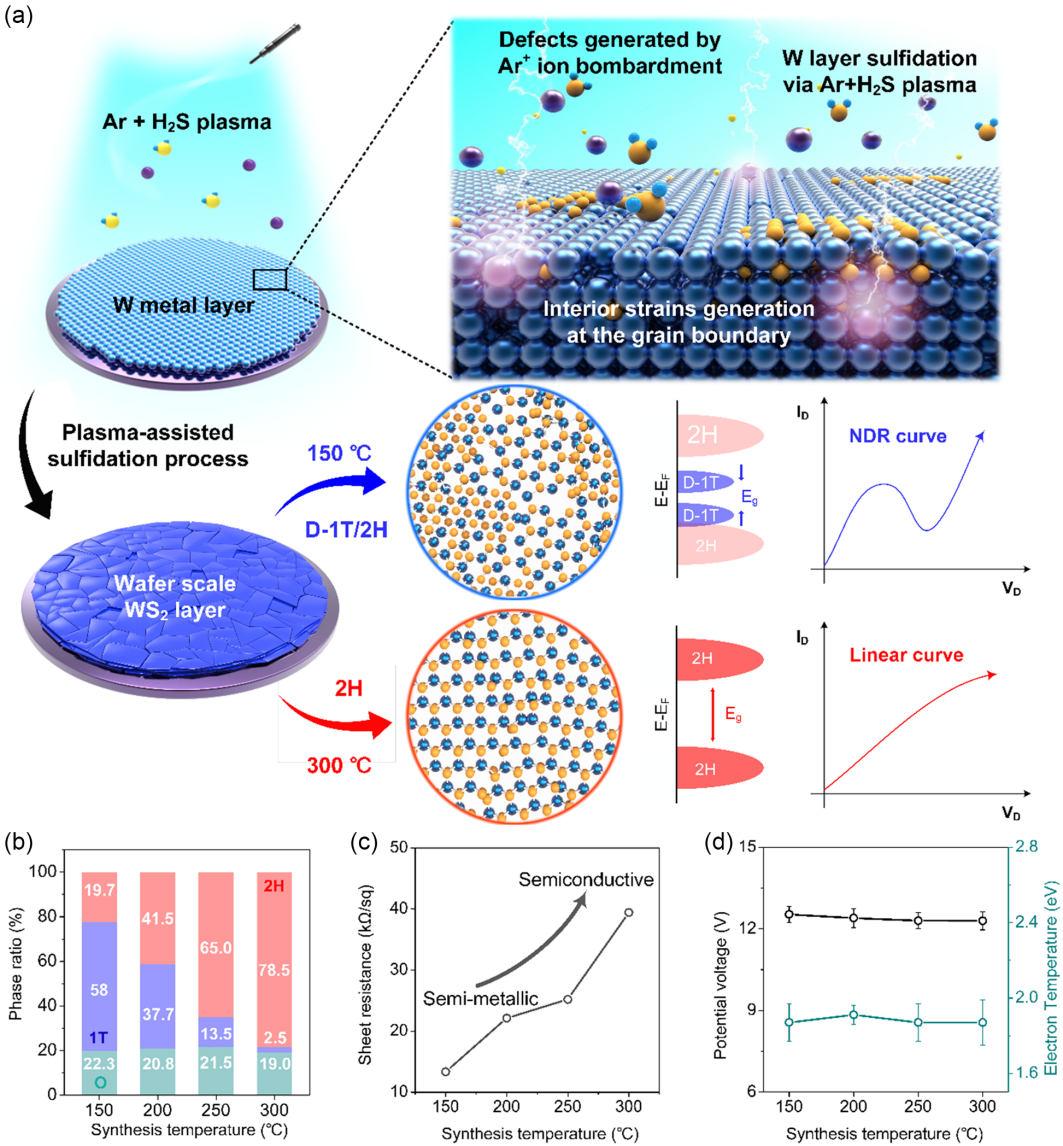
Overview of the phase modulation process. a) Schematic diagram of the formation of multiple‐phased tungsten disulfide (MP‐WS_2_) and 2 H WS_2_ thin film depending on the synthesis temperature via a plasma‐assisted metal‐layer sulfidation process and the corresponding shape of the current–voltage (*I–V*) electrical characteristic of the MP‐WS_2_/*p*‐Si heterostructure for each synthesis condition (blue curve: 150 °C, red curve: 300 °C). b–d) Temperature dependence: b) 1 T, 2 H, and oxidation X‐ray photoelectron spectroscopy (XPS) peak ratio and c) sheet resistance variation of the MP‐WS_2_ thin film. d) Potential voltage and electron temperature of Ar+H_2_S plasma depending on the synthesis temperature.

MP‐WS_2_ was prepared with a metal‐layer sulfidation process via inductively coupled plasma (ICP)‐type PE‐CVD (**Figure** **1**a). Photographs of the as‐synthesized WS_2_ showed that the MP‐WS_2_ layer on the 4 in SiO_2_/Si wafer showed good uniformity (Figure S1, Supporting Information). In contrast, unlike the conventional metal‐layer sulfidation process, the structural characteristic of the MP‐WS_2_ layer prepared with ICP‐type plasma was distinct owing to ionization at low temperatures. Large‐scale MP‐WS_2_ was fabricated by modulating the 2 H‐ to D‐1 T‐phase ratio in MP‐WS_2_ according to the synthesis temperatures from 300 to 150 °C. X‐ray photoelectron spectroscopy (XPS) was conducted to determine the elemental composition and oxidation state of the synthesized MP‐WS_2_ according to the temperature (Figure 1b and S2, Supporting Information). The two predominant peaks of W 4f_7/2_ and W 4f_5/2_ shifted gradually from 32.25 to 32.7 eV and 34.35 to 34.80 eV, respectively, as the temperature decreased (Figure S2, Supporting Information) .^[^ ^28^ ^]^ Figure 1b shows the polymorphic ratio of the D‐1 T phase to the 2 H phase as well as tungsten oxide in MP‐WS_2_ based on the XPS results. MP‐WS_2_ retained 58% of the D‐1 T phase exhibiting only 19.7% of the 2 H phase, whereas the 2 H phase increased to 78.5% at 300 °C. Hence, the synthesis temperature plays a crucial role in changing the lattice structure and regulating the amount of the D‐1 T phase. However, it does not give an influence on the formation of tungsten oxide, exhibiting the almost same percentage. The D‐1 T phase of MP‐WS_2_ synthesized at a temperature of 150 °C was maintained for 1 year under an ambient atmosphere with only accompanying oxidation because of the incorporated oxygen with the dangling bonds of W atoms, as evidenced by the W^6+^ peaks at 35.3 eV (W^6+^ 4f_7/2_) and 37.6 eV (W^6+^ 4f_5/2_) (Figure S3, Supporting Information).^[^ ^29^ ^]^ The as‐synthesized MP‐WS_2_ was stable because the phase transition from 1 T to 2 H was not observed under the external environment.^[^ ^30^ ^]^ Normal four‐probe measurements were taken to investigate the resistivity and a concomitant phase dependence (Figure 1c). The sheet resistance increased linearly from 13.3 at 150 °C to 39.4 kΩ sq^−1^ at 300 °C, indicating a transformation from semimetallic to semiconductive properties. The variation in resistance indirectly showed that the dominant phase in the MP‐WS_2_ changed from the semimetallic D‐1 T phase to the semiconducting 2 H phase as the temperature increased.^[^ ^31^ ^]^ The plasma condition plays a crucial role in the interaction between ions and materials, leading to a modification of their properties. Therefore, it is necessary to clarify the relationship between the plasma properties and temperature‐dependent phase control achieved through the plasma‐assisted sulfidation method. The electron temperature (*T*_e_) and plasma potential (*V*_p_) determined using Langmuir probe measurements were similar regardless of the synthesis temperature (Figure 1d). The plasma characteristics are not dependent on the synthesis temperature, but the thermodynamic components contributed to the phase control. Additional plasma diagnoses were carried out to investigate other plasma properties using optical emission spectroscopy (OES), retarding field energy analyzer (RFEA) in Figure S4 and Table S1, Supporting Information.

## 3 Material Characterization of the MP‐WS_2_

**Figure 2:**
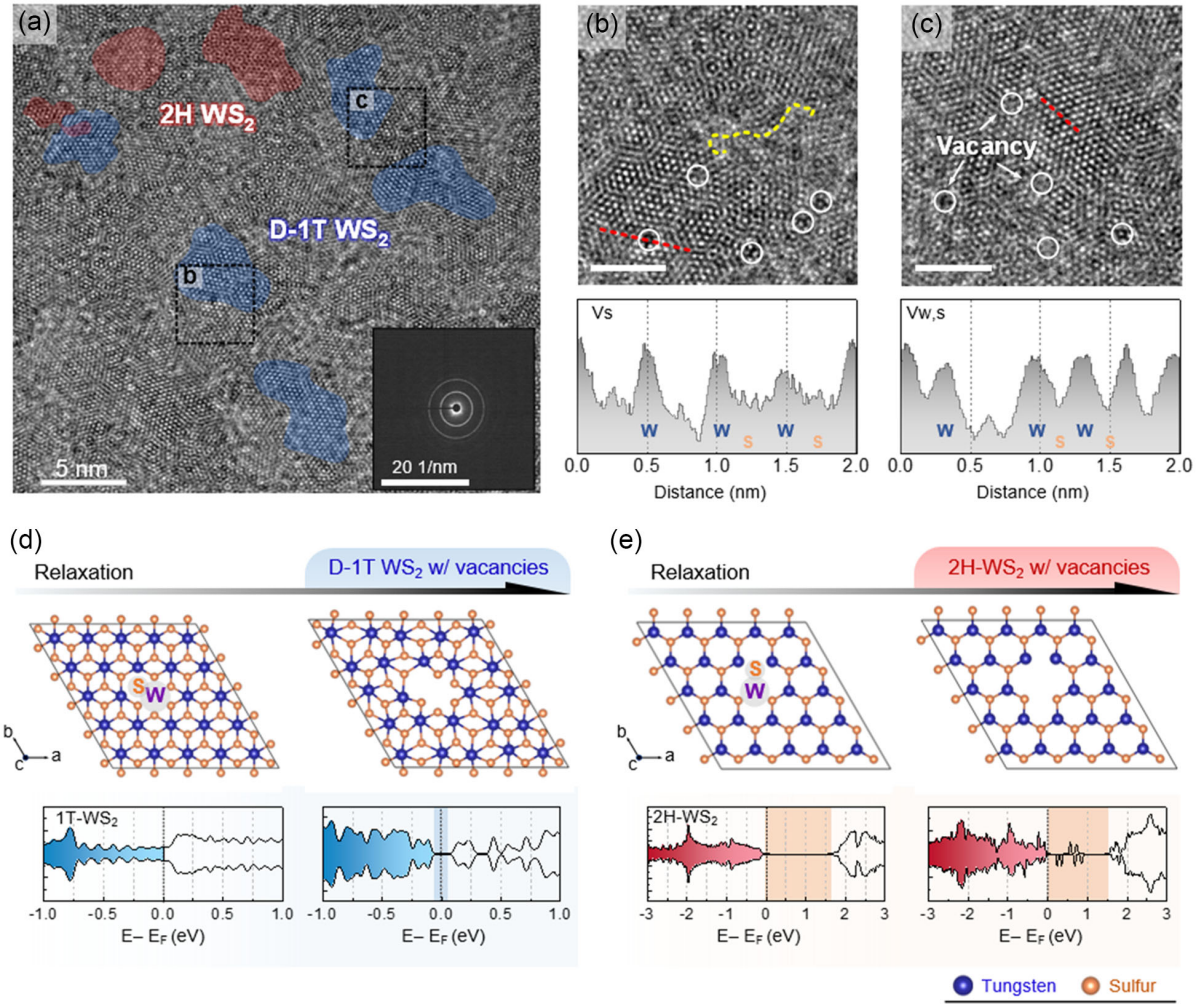
Calculation of the material characteristics of the defect accompanied D‐1 T/2 H WS_2_. a) Top‐view high‐resolution transmission electron microscopy (HR‐TEM) image of the MP‐WS_2_ thin film and corresponding selected area electron diffraction pattern (blue‐shaded region: 1 T phase; red‐shaded region: 2 H phase). b,c) Enlarged HR‐TEM images of regions where b) *V*_s_ and c) *V*_w,s_ are discovered in MP‐WS_2_ (scale bar: 2 nm); the bottom graphs exhibit intensity profiles (red‐dotted lines in the TEM images). The yellow circles indicate several kinds of vacancies or defects. The orange‐dotted line shows the grain boundary. d,e) Atomistic configuration and total density of states (DOS) results of d) 1 T WS_2_ without (left)/with (right) *V*_w,s_ and e) 2 H WS_2_ without (left)/with (right) *V*_w,s_ after structural relaxation; the vertical dash line at 0 eV indicates Fermi level (*E*_F_). Each colored domain in the DOS graph shows a bandgap between the valence and the conduction bands.

High‐resolution transmission electron microscopy (HR‐TEM) is an appropriate method to verify the lattice condition of the MP‐WS_2_ prepared via the proposed sulfidation mechanism. During the synthesis process, nucleation points in the metal layer interrupt grain growth resulting in a nanocrystalline regime. The structural features of MP‐WS_2_ fabricated at 150 °C for 1 h were examined by plan‐view HR‐TEM (**Figure** **2**a). Selected area electron diffraction revealed sub‐10 nm nanocrystals embedded in the WS_2_ layer, which is indicated by two distinct ring patterns corresponding to the (100) and (110) planes. This result agreed with the X‐ray diffraction (XRD) pattern of as‐synthesized WS_2_ samples, showing a broad peak below 30° corresponding to the (002) plane (Figure S5, Supporting Information). The broader peak indicates the smaller crystallinity based on the Scherrer equation. Furthermore, two types of lattice coordination, 1 T and 2 H phases, which are characterized by the octahedral and trigonal coordination of sulfur–tungsten–sulfur (S–W–S), were contiguous and adjoined together (Figure S6, Supporting Information). TEM (Figure S7a, Supporting Information) of the MP‐WS_2_ sample synthesized at 300 °C revealed the widespread trigonal coordination of S–W–S, corresponding to the predominant 2 H‐phase regime.

In contrast, defects and grain boundaries are commonly confirmed in both cases, caused by densely distributed nucleation points on the metal layer and Ar^+^ bombardment (Figure 2b,c and S7b,c, Supporting Information). Despite these dense defects and grain boundaries, the higher S/W atomic ratio over two was attributed to numerous nanosize grains, as shown in Figure S8, Supporting Information.^[^ ^32^ ^]^ Sulfidation at high temperatures reduces these defects.^[^ ^33^ ^]^ The line profiles along the red‐dotted line in the TEM images showed much lower intensity voids resembling spaces, such as S or W vacancies. Section S1–S3, Supporting Information, provide further details on the synthesis mechanism of the MP‐WS_2_ and its phase transition with varying temperatures.

Density‐functional theory (DFT) calculations were carried out to understand the electronic structure of the proposed MP‐WS_2_ involving several types of vacancies. Section S4, Supporting Information, describes the geometric information of the initial and relaxed structures. After DFT structural relaxation, 1 T WS_2_ with *V*_w,s_ became distorted because of inherent structural instability in the 1 T phase and lattice stress caused by the defects, while 1 H‐WS_2_ exhibited an intact hexagonal lattice, as shown in Figure 2d,e. Even when more defects were generated, 1 H‐WS_2_ still had the inherent structures, unlike the 1 T WS_2_ cases (Figure S13, Supporting Information). Furthermore, based on the total density of states (TDOS), pure 1 T WS_2_ has metallic properties, whereas D‐1 T WS_2_ transformed into a semimetal with a trivial bandgap of ≈ 0.1 eV. This was in accordance with previously reported research showing that MoS_2_ with point defect exhibited a semimetallic band structure.^[^ ^34^ ^]^ In the case of the 2 H WS_2_ samples, their bandgap did not change, but many trapped states in the bandgap were observed (Figure S14, Supporting Information). As a result, MP‐WS_2_ has a grooved band structure consisting of a semimetallic D‐1 T phase and a semiconducting 2 H phase.

## 4 Effect of Phase Modulation on an NDR Generation

**Figure 3:**
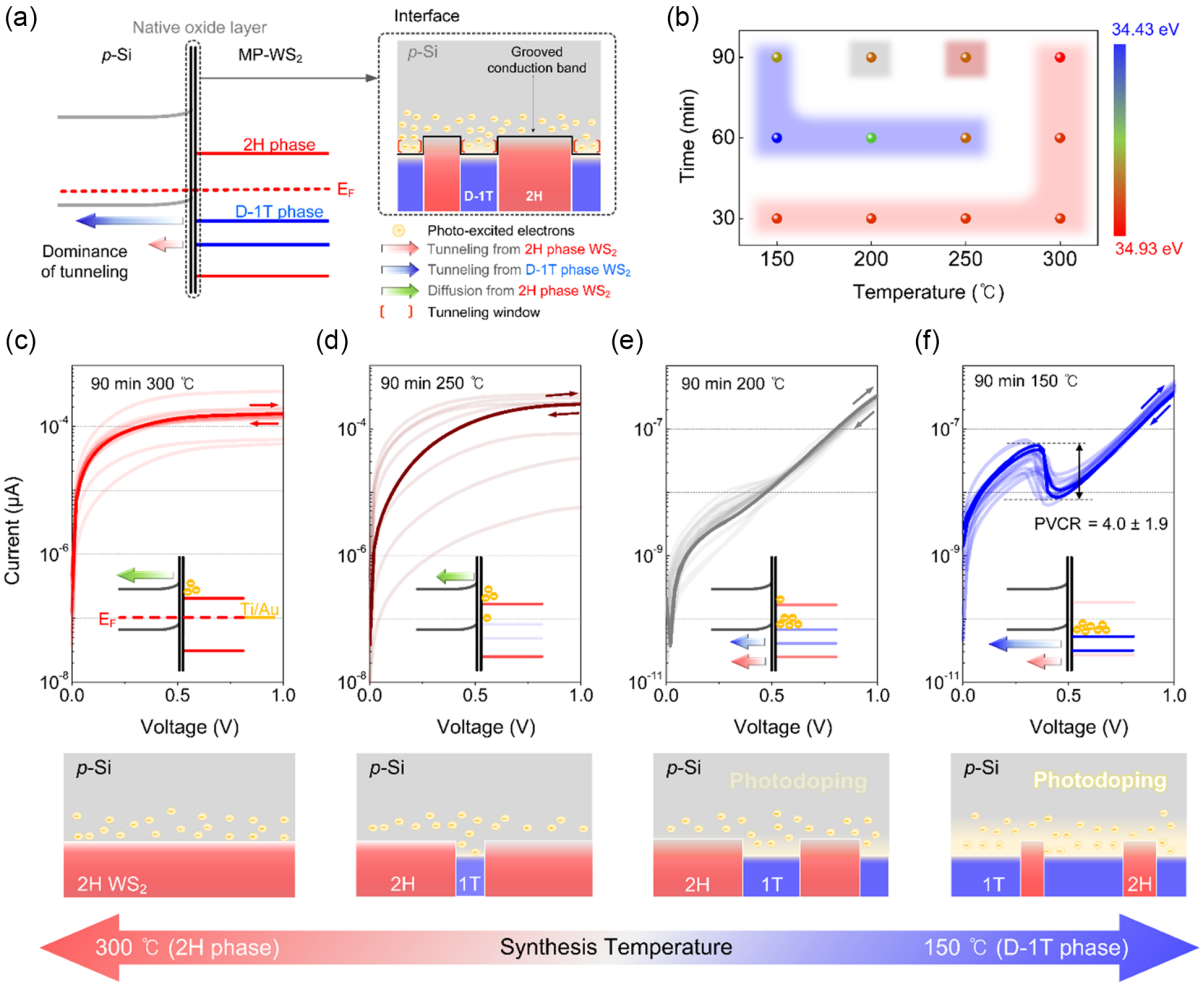
Mechanism analysis and optimization of the MP‐WS_2_/*p*‐Si heterostructure. a) Schematic diagram of electron transportation in the band alignment of MP‐WS_2_/*p*‐Si heterostructure. The inserted image shows the side view of the band alignment between the MP‐WS_2_ (grooved conduction band) and *p*‐Si (flat conduction band) at the junction interface. b) Mapping plot of XPS W 4f_5/2_ peak positions of the MP‐WS_2_ in relation to process temperature (150, 200, 250, and 300 °C) and process time (0.5, 1, and 1.5 h). The color of the blur indicates the shape of the *I–V* curve at each condition: c) red, d) brown, e) gray, and f) blue). c–f) Electrical characteristics of 10 MP‐WS_2_/*p*‐Si negative differential resistance (NDR) devices for each synthesis condition: process time, 1.5 h; synthesis temperature: c) 300 °C, d) 250 °C, e) 200 °C, and f) 150 °C. The inserted image and below image for each condition demonstrate the photoexcited electrons accumulation state in each band alignment.

*p*‐type bulk silicon shows a good synergistic effect with MP‐WS_2_ for band‐to‐band tunneling because it can generate large amounts of photoexcited electrons under an illumination state. Measurements of the electrical properties of the MP‐WS_2_/*p*‐Si heterostructure revealed distinctive current behavior variations under multiwavelength light illumination according to synthesis time and temperatures. The detailed device fabrication process is described in the Environmental Section. **Figure** **3**a presents the energy‐band alignment that elucidates the mechanism of photoexcited electrons tunneling via D‐1 T WS_2_. The detailed band properties of the 2 H WS_2_, D‐1 T WS_2_, and *p*‐Si were calculated, as shown in Figure S15 and Table S4, Supporting Information. Considering that the D‐1 T WS_2_ (*E*_g_  = 0.49 eV) is aligned parallel with 2 H WS_2_ (*E*_g_ = 1.48 eV), the MP‐WS_2_ eventually forms a grooved conduction band structure, as shown in the inserted image in Figure 3a. When multiwavelength light illuminates the device, photoexcited electrons generated via a photoelectric effect from *p*‐Si accumulate on the grooved conduction bands of the MP‐WS_2_. The electrons are then transported through different routes according to the type of band alignment between two different WS_2_ phases and *p*‐Si, respectively. In the case of 2 H WS_2_/*p*‐Si, the normal type‐II heterostructure only allows the thermionic current from the conduction band of 2 H WS_2_ to *p*‐Si along the applied voltage. For D‐1 T WS_2_, the proper band characteristics and minuscule bandgap are suitable for building a broken gap with *p*‐Si, promoting band‐to‐band tunneling preferentially. In other words, the predominance of the D‐1 T phase in MP‐WS_2_ is sufficient to convert net electron transportation from thermionic diffusion to band‐to‐band tunneling. In addition, photoexcited electron accumulation on the conduction band of D‐1 T WS_2_ contributes to shift Fermi level to the conduction band as a “photodoping effect”, resulting in apparent NDR behavior, as shown in Figure 3a.

In this study, band‐to‐band tunneling was optimized by plotting the correlation between the synthesis parameters (process times: 0.5, 1.0, and 1.5 h; process temperatures: 150, 200, 250, and 300 °C) and the XPS W 4f_5/2_ peak positions (Figure 3b). The blurred colors present the corresponding current behavior of the MP‐WS_2_/*p*‐Si devices in Figure 3c–f using the same color. Figure S16, Supporting Information, shows the measured electrical characteristics of the devices prepared at 0.5 h. The blurred color distribution under each condition showed that the current behavior strongly depends on the W 4f_5/2_ peak position. Hence, the predominant phase in the WS_2_ strongly influences the major electron transportation regime for the NDR device. Under multiwavelength light illumination (5.3 mW cm^−2^), the light current of the device prepared at 300 °C and 1.5 h shows a linear curve with 352.7 μA at a *V*_D_ of 1 V, indicating a 35‐fold higher current than that of the *p*‐Si (10.0 μA at a *V*_D_ of 1 V) (Figure 3c). The large current can be achieved by the conduction band of 2 H WS_2_ between silicon and the electrode, alleviating the Schottky barrier (Figure S17, Supporting Information). In contrast, the thermionic current decreased gradually when the portion of the D‐1 T phase increased in the MP‐WS_2_ as the temperature decreased to 200 °C (Figure 3d). Consequently, the electron transportation from 2 H‐phase WS_2_ shrank (Figure 3e). Finally, owing to sufficient D‐1 T phase in the MP‐WS_2_, the device at 150 °C exhibits remarkable NDR behavior with a maximum peak current of 66.9 nA and a peak‐to‐valley current ratio (PVCR) of 6.7, as shown in Figure 3f. Section S5, Supporting Information, describes the overall symmetric NDR *I–V* curve operating mechanism in detail.

**Figure 4:**
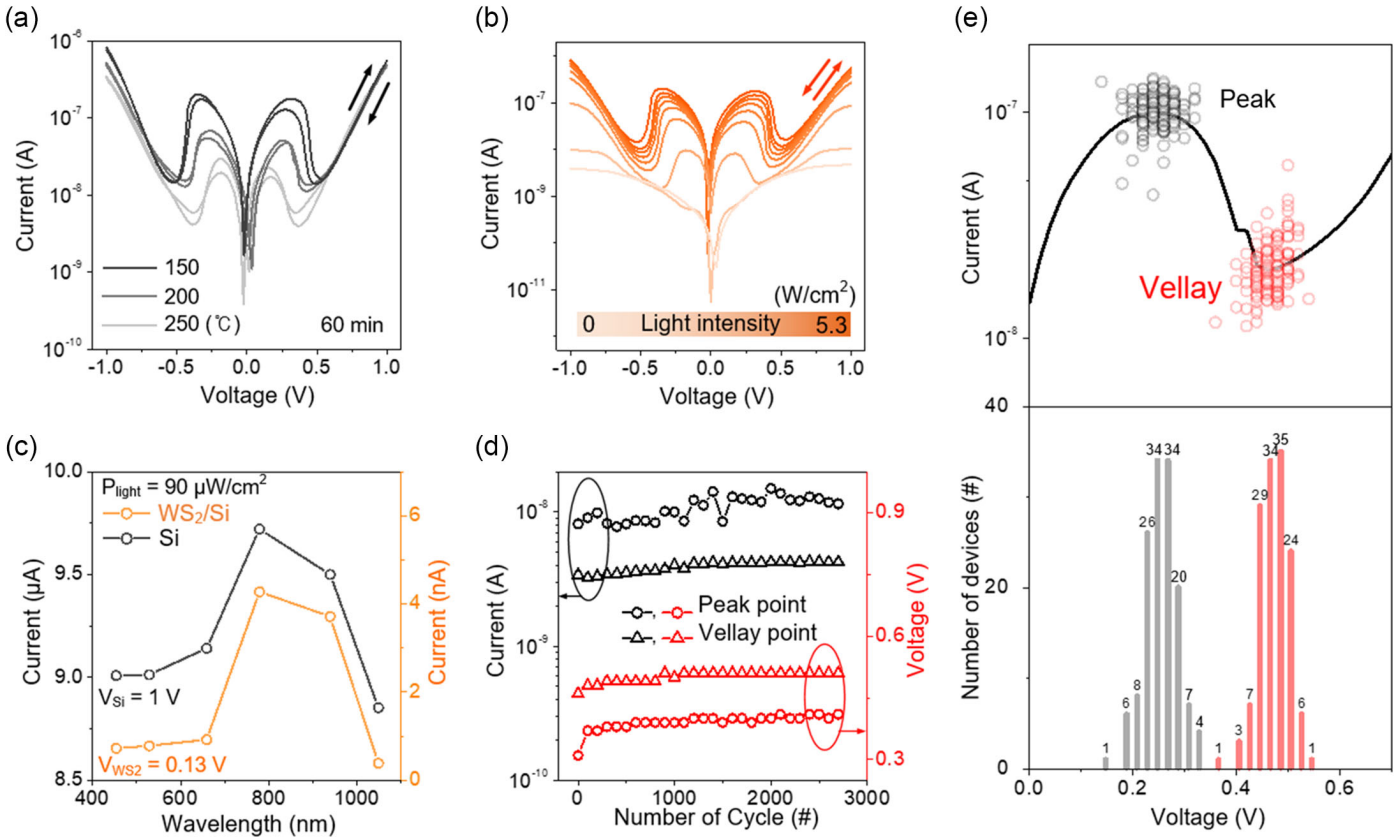
Verification of NDR performance and reliability of the MP‐WS_2_/*p*‐Si device. a) Comparison of the current behavior of the MP‐WS_2_/*p*‐Si diode according to the synthesis temperature from 250 to 150 °C. b) The electrical characteristics depending on the illuminated light intensity on the device (multiwavelength light intensity condition: from 0 to 5.3 mW cm^−2^). c) Photoresponsivity analysis of the MP‐WS_2_/*p*‐Si and single *p*‐Si diode for each light wavelength condition (monochromatic light wavelength condition: 455, 530, 660, 780, 940, and 1050 nm with an intensity of 90 μW cm^−2^). d) The endurance performance of the device for 2700 cycles at the peak and valley point (dark circle: peak/valley current; red circle: peak/valley voltage). e) Uniformity test for the peak and valley points distribution of the proposed 140 devices.

The temperature‐dependent variations in the *I–V* curves of the MP‐WS_2_/*p*‐Si heterostructure synthesized for 1 h also showed the importance of the D‐1 T phase to induce efficient electron tunneling, as shown in **Figure** **4**a. As the synthesis temperature decreased from 250 to 150 °C, there was a gradual increase in the peak current and valley voltage, eventually reaching a maximum current of 0.21 μA and shifting the valley voltage from 0.3 to 0.5 V. Ultimately, the NDR device of MP‐WS_2_ at 150 °C and 1 h was optimized with a peak‐to‐valley ratio of 13.8, indicating superior performance compared to previous studies, as shown in Table S5 and Figure S19, Supporting Information. In addition to the role of the D‐1 T phase, light illumination is another determinant for driving the NDR phenomenon. The electrical characteristics of the optimized NDR device depending on the illuminated light intensity (multiwavelength light intensity condition: from 0 to 5.3 mW cm^−2^) were investigated to reveal the contribution of photoexcited electrons in the improved NDR behavior, as shown in Figure 4b. In contrast, the linear *I–V* curve was confirmed in the dark; the NDR tendency gradually becomes apparent with an increase in light intensity, showing tremendous current growth from 8 × 10^−10^ to 2 × 10^−7^ A at the peak point. Hence, the photogating effect originated from photoexcited electrons and eventually constructed the broken gap at the D‐1 T WS_2_/*p*‐Si interface. Figure 4c presents the photoresponsivity of the MP‐WS_2_/*p*‐Si and single *p*‐Si diode under different monochromatic light wavelength conditions (namely 455, 530, 660, 780, 940, and 1050 nm) with a constant intensity of 90 μW cm^−2^. The trajectory of the light current of the MP‐WS_2_/*p*‐Si followed a similar pattern to that of the Si photodiode, indicating that the photoexcited electrons are supplied by *p*‐Si.^[^ ^35^ ^]^ The reliability of the NDR device using the MP‐WS_2_/*p*‐Si heterostructure was evaluated under an ambient atmosphere. NDR behavior of the proposed MP‐WS_2_/*p*‐Si heterostructure remains for 2700 cycles. At the peak point, current and voltage value demonstrate variation during the cycling test. Oxidation at the grain boundary and several defects in the MP‐WS_2_ layer provide many trap sites that can induce fluctuation in tunneling transportation. However, since the WS_2_ layer possesses only 5 nm thickness, it is too thin to influence sufficiently in NDR generation (valley current, 3.93 ± 0.34 nA; peak current, 10.85 ± 2.13 nA; valley voltage, 501 ± 13 mV; peak voltage, 391 ± 19 mV) (Figure 4d). The long‐term stability result showed superior performance sustainability, of which the peak/valley positions remained for 122 days (Figure S20, Supporting Information). Moreover, 140 NDR devices on centimeter‐scale MP‐WS_2_/*p*‐Si were selected randomly to estimate the uniformity of the performance. As a result, identical performance was obtained with comprehensive current/voltage deviation (valley current, 20.86 ± 6.30 nA; peak current, 101.42 ± 17.52 nA; valley voltage, 467 ± 29 mV; peak voltage, 247 ± 32 mV) (Figure 4e). Additional current and PVCR distribution figures are posted in Figure S21, Supporting Information.

## 5 Application in Photoreactive RAM

**Figure 5:**
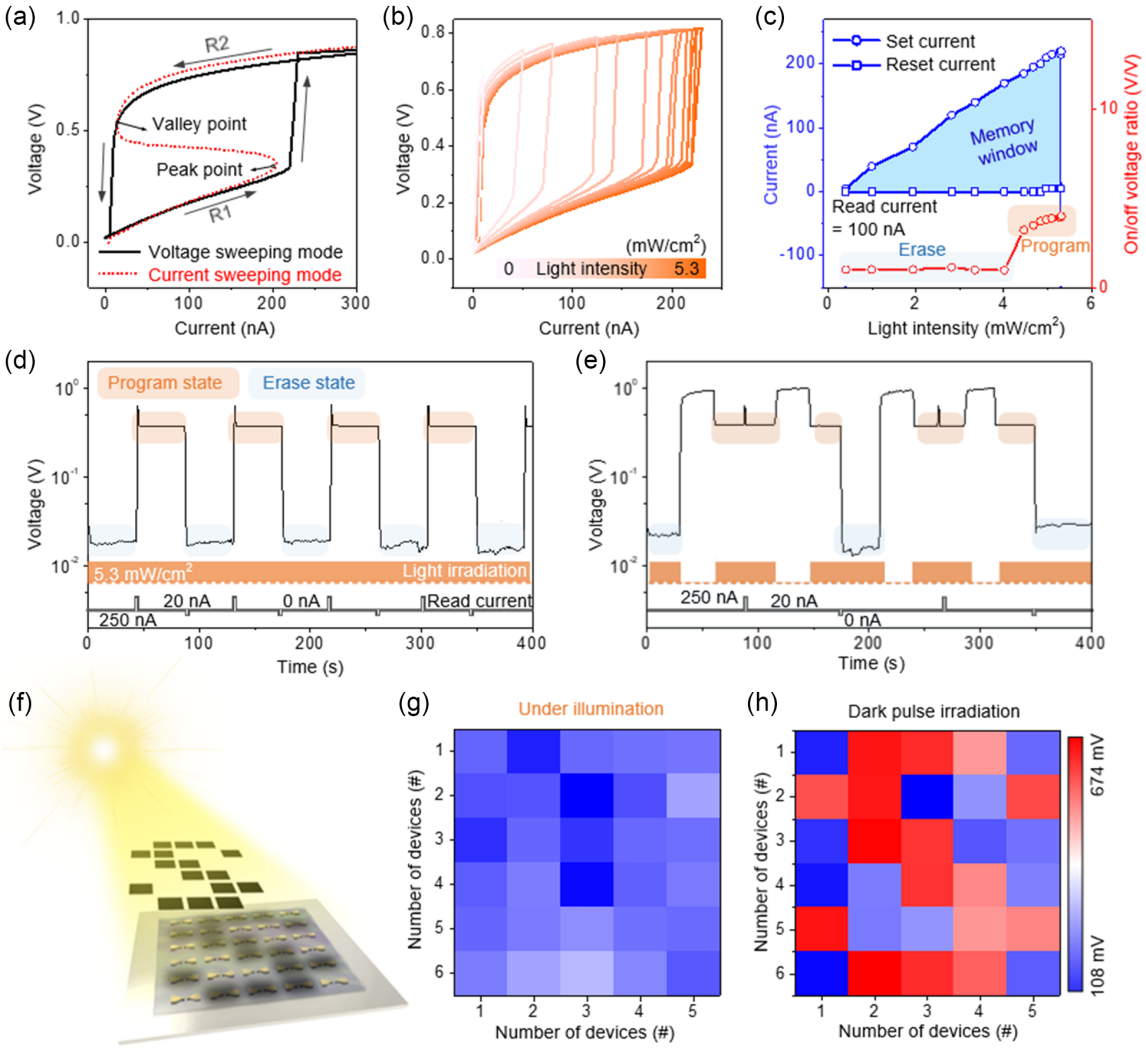
Implementation of photoreactive random‐access memory (RAM) application via the proposed NDR device. a) Mechanism of the photoreactive RAM under current‐sweeping mode. b) Memory performance evaluation of the MP‐WS_2_/*p*‐Si under light intensity variation from dark to 5.3 mW cm^−2^. c) Variation in the memory window and memory operation state at a read current of 100 nA and a change in light intensity from dark to 5.3 mW cm^−2^. d,e) Memory retention of the photoreactive RAM operated with d) current pulse and e) current pulse with dark pulse. f,g) Schematic diagram of photoreactive memory performance test using the NDR device; g) fully illumination state and h) after illuminating dark pulse on the devices located specific position for 1 s.

The resistive bistability is suitable for utilizing the NDR device as a RAM application with current‐sweeping mode (**Figure** **5**a). Given the forward current sweeping to 300 nA under multiwavelength light illumination, the output voltage is transited from the R1 to R2 state after passing the peak point. In contrast, the output voltage settles from the R2 state to the R1 state after the valley point when the current is swept backward from 300 to 0 nA. Consequently, the *I–V* loop allows the MP‐WS_2_/*p*‐Si heterostructure to be used as RAM operation. The photoreactivity of the RAM devices with multiwavelength light intensities from 0 to 5.3 mW cm^−2^ was evaluated, as shown in Figure 5b. As the intensity of multiwavelength light gradually weakens, the memory window narrows in proportion to the tunneling current, decreasing the resistive bistability. This excellent memory window tunability enables control of the program/erase state depending only on the light intensity at a fixed read current (Figure 5c).

The device operation range was also extended linearly from 0–5 nA to 5–220 nA as the illumination was increased to 5.3 mW cm^−2^. At a read current of 100 nA, the NDR device showed an erase state within a light intensity of 4 mW cm^−2^, presenting an ON/OFF voltage ratio of 1 V V^−1^. In contrast, the ON/OFF voltage ratio exceeded 3 V V^−1^ when the light intensity was more than 4 mW cm^−2^. The retention characteristics were estimated to confirm the performance of the photo‐assisted programmable RAM by controlling the current pulse and illumination state (Figure 4d,e). The programming and erasing operations are implemented with a positive current pulse of 250 nA and a negative current pulse of 0 nA, respectively. In addition, the memory state was recognized at a read current of 20 nA. Under continuous light illumination, when a positive current pulse of 1 s width was applied to the device, the memory state of the device was converted from “erase state” to “program state” with an ON/OFF voltage ratio of 40 V V^−1^. In contrast, the memory state returns to the “erase state” with an applied negative current pulse, exhibiting perfect memory state control (Figure 5d). Figure 5e shows the effect of applying a dark pulse on the device, which initializes the memory state to the “program state” regardless of the previous state. The result suggests diverse functionality of the proposed device according to electrical and optical signals.

In this study, 5 × 6 photoactive RAM devices were fabricated using the MP‐WS_2_/*p*‐Si heterostructure to validate their practicality, as shown in Figure 5f and S22, Supporting Information. A dark pulse was implanted selectively by shading for 1 s to generate an optical program state. Initially, all the devices remained erased under illumination, indicating a low voltage distribution of 0.220 ± 0.062 V with a read current of 20 nA (Figure 5g). In contrast, when injecting dark pulse on the devices located along the shape of the character “S”, the voltage distribution on the shaped region increased to 0.601 ± 0.051 V under the same read current (Figure 5h). The device exhibited discriminable memory operation mode depending on the light intensity. These memory retentions and 5 × 6 device array tests confirmed the feasibility of novel photoreactive RAM using a large‐area MP‐WS_2_/*p*‐Si NDR device array as an electrical and optical data storage.

## 6 Conclusion

A plasma‐assisted sulfidation process was used to obtain optimal NDR devices of the MP‐WS_2_/*p*‐Si heterostructure by directly controlling the D‐1 T/2 H‐phase ratio. The amount of D‐1 T phase in the WS_2_ thin film was associated with the synthesis temperature rather than plasma properties based on the plasma diagnosis and material characterization. Furthermore, DFT calculations and empirical measurements supported the electronic structure of MP‐WS_2_, the formed grooved conduction band, which consisted of a semiconductive 2 H phase and semimetallic D‐1 T phase. The MP‐WS_2_ and *p*‐Si heterostructure provided the proper electronic structure for generating NDR phenomena because of the following two factors: 1) semimetallic D‐1 T‐phase WS_2_ for achieving band‐to‐band tunneling and 2) enhancement of the tunneling probability enhancement via effective photogating effect. Consequently, under the optimized condition of 150 °C and 1 h, the D‐1 T phase occupied a 77.4% area in the MP‐WS_2_, and the NDR device exhibited a large PVCR value of 13.8 with a high light current of 0.21 μA under multiwavelength light illumination. The stability for 2700 cycles and high‐performance uniformity demonstrated the sustainability of the MP‐WS_2_ thin film and the reliability of the NDR device. Finally, the writing and erasing operations according to the electrical and optical signals represented the practicality of the MP‐WS_2_/*p*‐Si heterostructure as a functional RAM application. The plasma‐assisted sulfidation process showed great potential for phase engineering as a one‐step synthesis method and opened a way to develop an advanced electronic application based on the D‐1 T phase.

## 7 Experimental Section

7.1

7.1.1

##### Synthesis of MP‐WS_2_ and 2 H WS_2_ via Plasma Enhanced Chemical Vapor Deposition Process

MP‐WS_2_ and 2 H WS_2_ were synthesized using a procedure reported elsewhere.^[^ ^36^ ^]^ The W metal thin film with a thickness of 1 nm was deposited uniformly onto a 4 in SiO_2_/Si substrate using an E‐beam evaporator. The W metal thin film underwent a H_2_ plasma treatment to eliminate the native oxide and contamination. The sulfurization plasma with an Ar and H_2_S mixed gas (v/v = 1/1) was performed to fabricate the MP‐WS_2_ composite under a pressure of 50 mTorr. The phase ratio between D‐1 T and 2 H was controlled by varying the process temperature (150, 200, 250, and 300 °C) and treatment time (0.5, 1, and 1.5 h). A radio frequency generator operated at the standard frequency of 13.56 MHz was used to ignite the plasma under a constant power of 550 W.

##### Fabrication of the MP‐WS_**2**_ **/p‐Si Photodiode**

The MP‐WS_2_/*p*‐Si photodiode was fabricated using the conventional PMMA‐assisted transfer method to transfer the synthesized MP‐WS_2_ layer to a boron‐doped 4 in *p*‐Si wafer. The *p*‐Si wafer (purchased from SemiRoad Co. Ltd., Korea) had a resistivity of 1–10 Ω·cm. The electrodes were patterned via a liftoff type on the MP‐WS_2_ layer using a conventional photolithography process. The MP‐WS_2_ layer was coated with photoresist (AZ 5214 E, Merck Performance Materials GmbH) before negative patterning. The electrodes were formed by consecutively depositing Au/Ti up to a thickness of 20/5 nm using E‐beam evaporation.

##### Material Characterization

The chemical bond information of the samples was confirmed by XPS (Thermo Fisher ESCALAB 250 Xi) with monochromatic Al Kα X‐rays at room temperature. The recorded W 4f and S 2p spectra were calibrated against the C 1s peak (reference at 284.6 eV) to determine the accurate binding energy. The surface morphology was analyzed by atomic force microscopy (AFM, NX10, Park System) in noncontact mode. A thin‐film XRD instrument (Smartlab, Rigaku) was used to examine the crystallinity of the WS_2_ thin film. Raman spectroscopy (Alpha300 M+, WITec GmbH) was performed with an excitation wavelength of 532 nm. The structural characterization of all samples was examined by HR‐TEM (JEM‐2100 F, JEOL) with an operating voltage of 200 kV. Cross‐sectional TEM images were obtained from the TEM samples using a focused ion beam (NX2000, Hitachi Ltd.).

##### Plasma Characterization

The ion energy distribution function was investigated using an RFEA (Impedans Ltd.) to determine the incident ion energy during the plasma with an energy resolution of 0.2 V and a time resolution of 5 s. A Langmuir probe (Hiden Analytical Ltd.) was used to obtain the plasma parameters (*V*_p_ and *T*_e_) calculated from the measured *I–V* curves. A variable voltage from −20 to 30 V was supplied to the platinum tip with a length and radius of 10 and 0.075 mm, respectively. The two diagnosis systems were grounded well and conducted to diagnose the electrical properties at various synthesis temperatures. The elemental composition of the plasma was analyzed by OES (Avantes, Avaspec‐2048).

##### Computational Details

All DFT simulations were performed to obtain the TDOS, as implemented in the Quantum Espresso packages.^[^ ^37^ ^]^ The generalized gradient approximation of the Perdew–Burke–Ernzerhof functional was used for electron exchange and correlation energy.^[^ ^38^ ^]^ The projector‐augmented wave pseudopotential was adopted for the electron–ion interaction. The supercell (5 × 5 × 1) dimension, including 25 W atoms and 50 S atoms, was 9.84 × 9.84 × 20 Å along with a vacuum space of 15 Å to avoid an interaction from the other slab. Γ‐centered Monkhorst–Pack k‐point mesh of 4 × 4 × 1 and 16 × 16 × 1 was set to sample the Brillouin zone for atomic relaxation and density of states calculations, respectively. The kinetic energy cutoff for the electronic wave expansion was set to 60 Ry. The Gaussian smearing method was adopted with a broadening of 0.001 Ry. The atomic position was fully relaxed until the convergence criterion on the forces was less than 10^−4^ Ry Bohr^−1^ and the energy criterion was less than 10^−8^ Ry by applying the Broyden–Fletcher–Goldfarb–Shanno algorithm.^[^ ^39^ ^]^

## Conflict of Interest

The authors declare no conflict of interest.

## Supporting information

Supplementary Material

## Data Availability

The data that support the findings of this study are available from the corresponding author upon reasonable request.
