# Supplementary material for: Temperature‐Dependent Phase Transition in WS2 for Reinforcing Band‐to‐Band Tunneling and Photoreactive Random Access Memory Application
Source: Small Sci. 2023 Nov 21;4(2):2300202. doi: 10.1002/smsc.202300202 (PMC11935019; doi:10.1002/smsc.202300202)
Supplement: Supplementary file 1 — Supplementary Material [file SMSC-4-2300202-s001.pdf]

Supplementary Information

Temperature Dependent Phase Transition in WS_2 for Reinforcing Band-to-band Tunneling and Photoreactive Random Access Memory Application

Gunhoo Woo[+], Jinill Cho[+], Heejung Yeom, Min Young Yoon, Geon Woong Eom, Muyoung Kim, Jihun Mun, Hyo Chang Lee, Hyeong-U Kim, Hocheon Yoo[*], and Taesung Kim[*]

[+]These authors contributed equally to this work.

This PDF file includes:

Figure S1. 4-inch wafer scale MP-WS_2 layer.
Figure S2. Temperature dependence XPS spectra of the MP-WS_2 thin film.
Figure S3. XPS spectra of the MP-WS_2 thin film (Pristine 150/300 ℃ and one year 150 ℃).
Figure S4. Plasma diagnosis results depending on synthesis temperature.
Figure S5. XRD patterns of the MP-WS_2 thin film.
Figure S6. Enlarged HR-TEM top-view image of the MP-WS_2 synthesized at 150 ℃.
Figure S7. HR-TEM images of MP-WS_2 synthesized at 300 ℃.
Figure S8. Temperature dependence S/W atomic ratio variation of the MP-WS_2 thin film.
Figure S9. Illustration of the plasma-assisted sulfidation process.
Figure S10. Plasma-assisted sulfidation process with a Faraday cage.
Figure S11. Investigation of the thickness variation in W metal layer and the MP-WS_2 thin film.
Figure S12. Atomistic configuration of 1T WS_2 and 2H WS_2.
Figure S13. Atomistic configuration of 1T and 2H WS_2 with vacancies.
Figure S14. Total density of states of 1T and 2H WS_2 depending on several vacancies.
Figure S15. Electronic structure of the MP-WS_2.
Figure S16. *I-V* curve of the MP-WS_2/*p*-Si at 0.5 hours and 150 to 300 ℃.
Figure S17. Comparison of the photocurrent of the 2H WS_2/*p*-Si with *p*-Si photodiode.
Figure S18. Operation mechanism of the photo-reactive NDR device.
Figure S19. Performance comparison of the proposed device with previous research.
Figure S20. Long-term stability test of the MP-WS_2/*p*-Si heterostructure.
Figure S21. Current and PVCR distribution of the 140 proposed NDR devices
Figure S22. Image of a 5 × 6 NDR devices array.
Supplementary information 1. Plasma-assisted synthesis process.
Supplementary information 2. Discussion on structural properties of MP-WS_2.
Supplementary information 3. Overall mechanism of plasma-assisted MP-WS_2 synthesis.
Supplementary information 4. Structural and electronic properties using DFT calculation.
Supplementary information 5. Discussion on NDR operation mechanism.
Table S1. Plasma characteristics for various powers.
Table S2. Atomic configuration of initial structures for pristine 1T/2H WS_2.
Table S3. Atomic configuration of relaxed structures for 1T/2H WS_2 with vacancies.
Table S4. Band characteristic of MP-WS_2 prepared at 150, 300 ℃ and *p*-Si.
Table S5. Performance comparison of the proposed device with previous research.

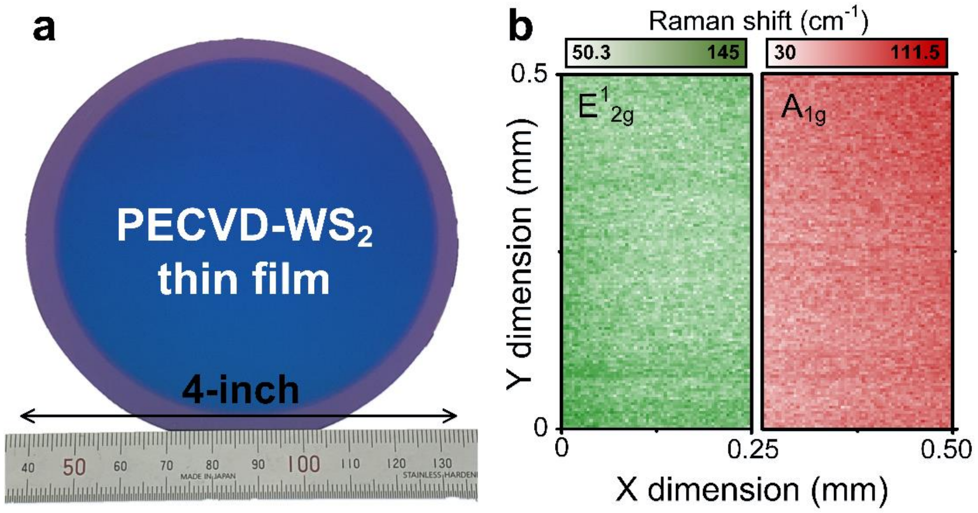

Figure S1. a) Optical image of 4-inch wafer scale MP-WS_2 layer. b) Raman mapping of the MP-WS_2 layer corresponding to the E^1_{2g} peaks and A_{1g} peaks at a 532 nm excitation laser.

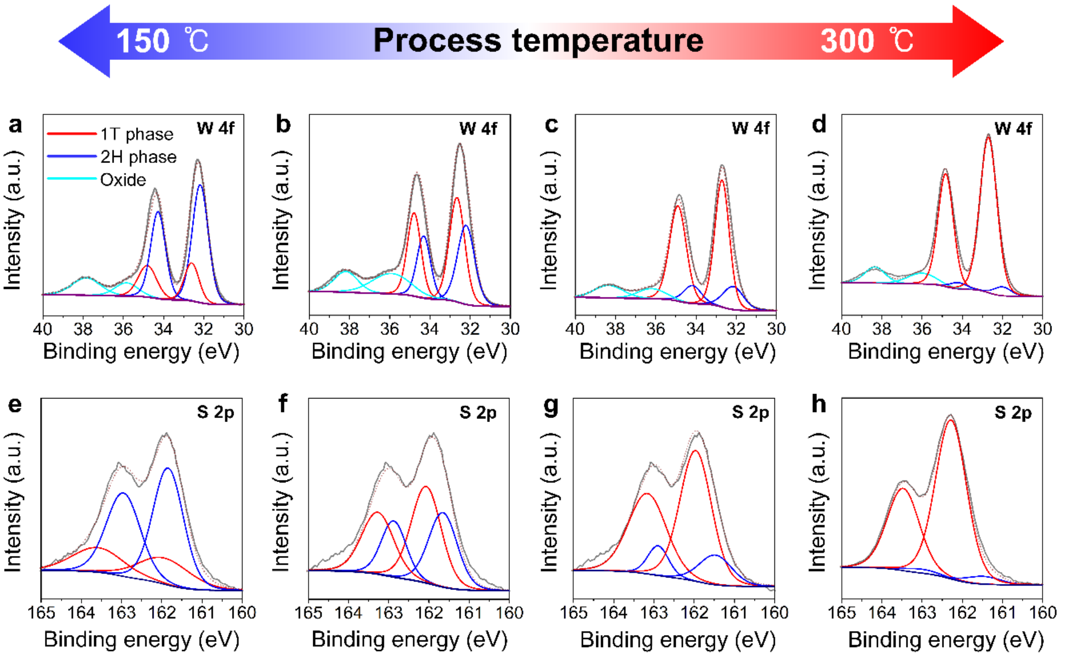

Figure S2. Temperature dependence XPS spectra of the MP-WS_2 thin film. High resolution XPS spectra of W 4f-core level for MP-WS_2 synthesized at a) 150 °C, b) 200 °C, c) 250 °C, and d) 300 °C, and S 2p-core level synthesized at e) 150 °C, f) 200 °C, g) 250 °C, and h) 300 °C.

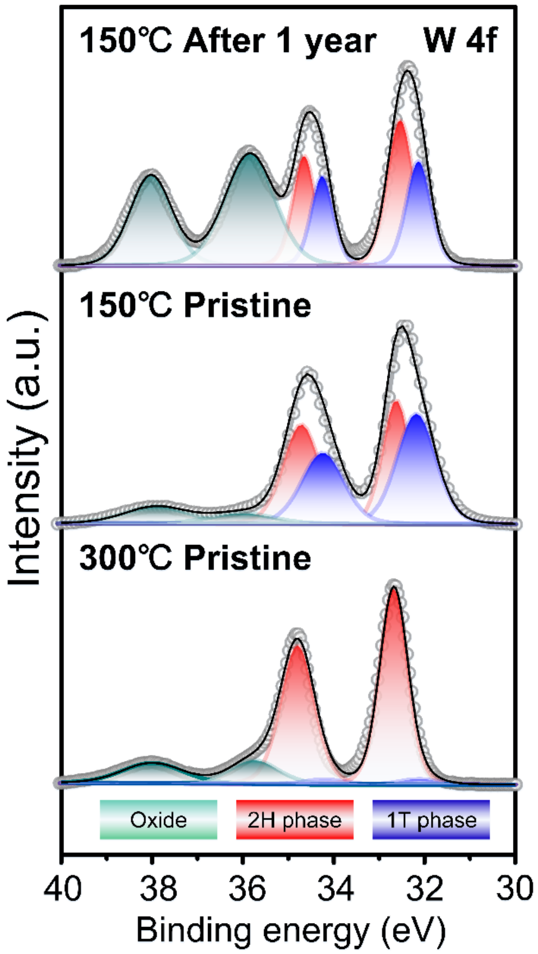

Figure S3. W 4f XPS spectra of the MP-WS_2 thin film. The MP-WS_2 samples were synthesized at (bottom) 300 °C and (middle) 150 °C. The top profile shows W 4f XPS spectra of one-year-aged MP-WS_2 prepared at 150 °C.

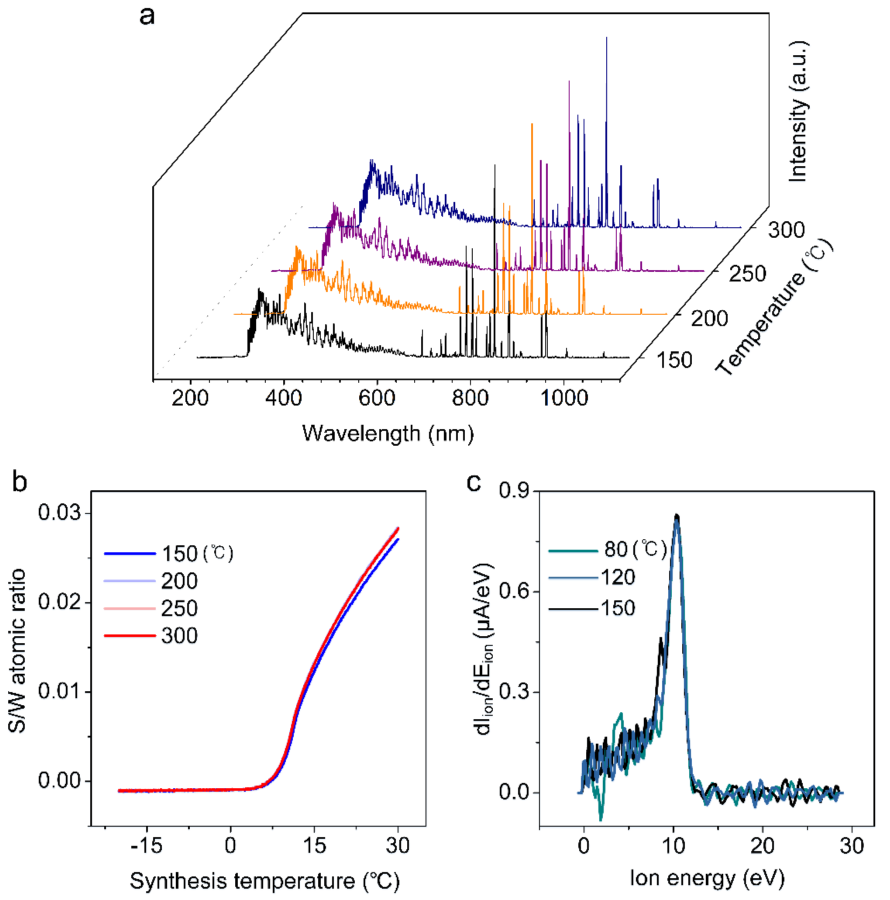

Figure S4. Plasma diagnosis results depending on synthesis temperature. a) OES result, b) Langmuir probe measurement, and c) RFEA result are plotted according to synthesis temperature from 150 °C to 300 °C.

Table S1. Plasma characteristics at various temperatures.

Power [W]	550			
Pressure [mTorr]	50			
Temperature [°C]	150	200	250	300
T_e [eV]	1.87	1.91	1.87	1.88
V_f [V]	7.1	6.87	6.89	6.84
V_p [V]	12.5	12.4	12.3	12.3

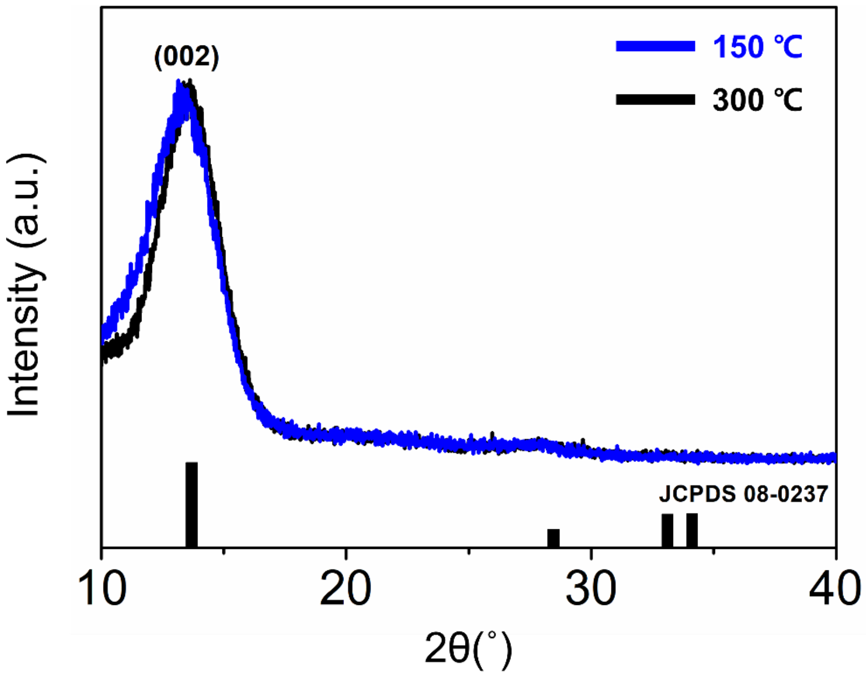

Figure S5. XRD patterns of the MP-WS_2 thin film.

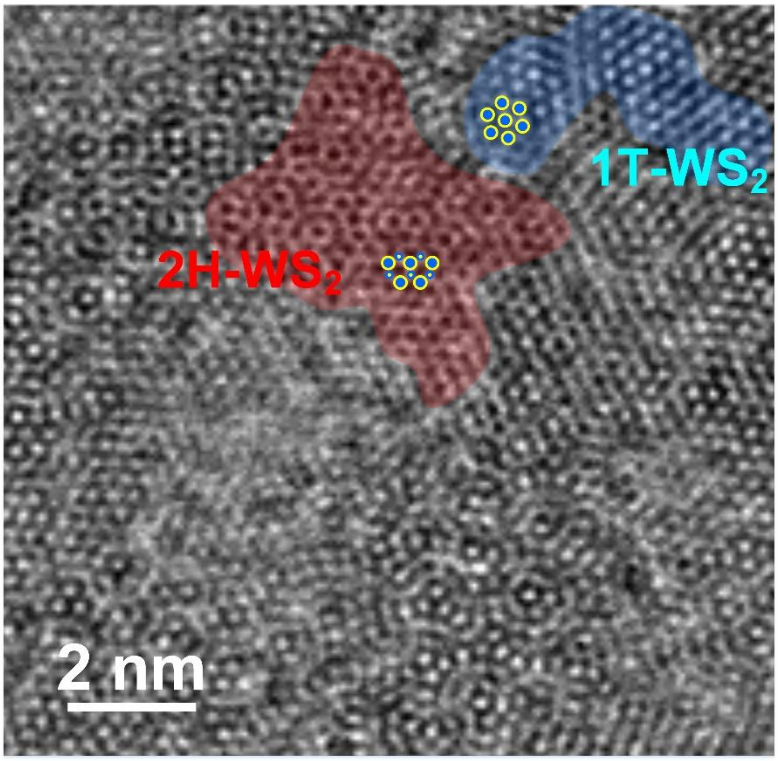

Figure S6. Enlarged HR-TEM top-view image of the MP-WS_2 synthesized at 150 °C. The blue and red shaded regions correspond to 1T WS_2 and 2H WS_2, respectively.

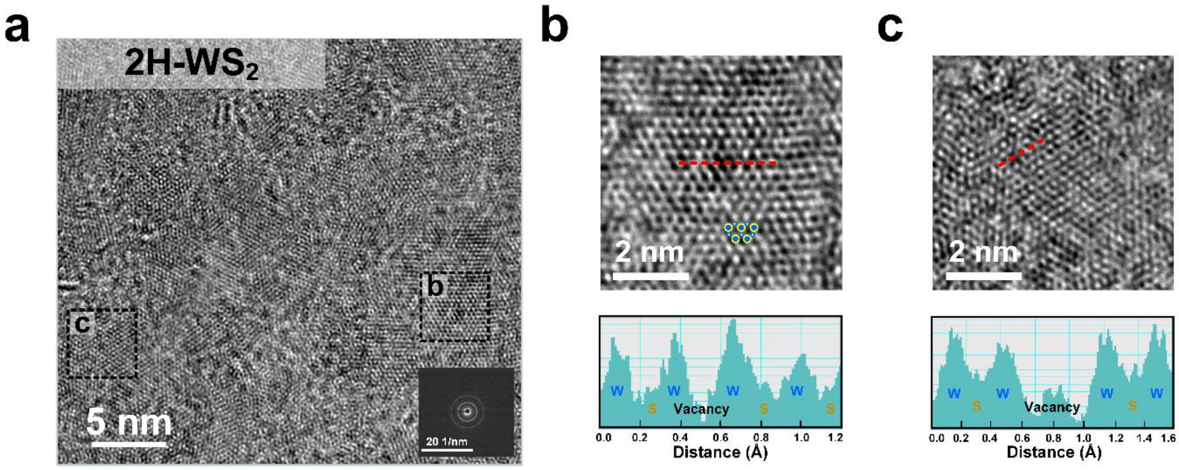

Figure S7. HR-TEM images of MP-WS_2 synthesized at 300 ℃. a) HR-TEM image of 2H WS_2, HR-TEM images of regions where b) V_s and c) $V_{s,w}$ were observed in the 2H WS_2. The bottom graphs exhibit the intensity profiles along the yellow dotted lines in the TEM images.

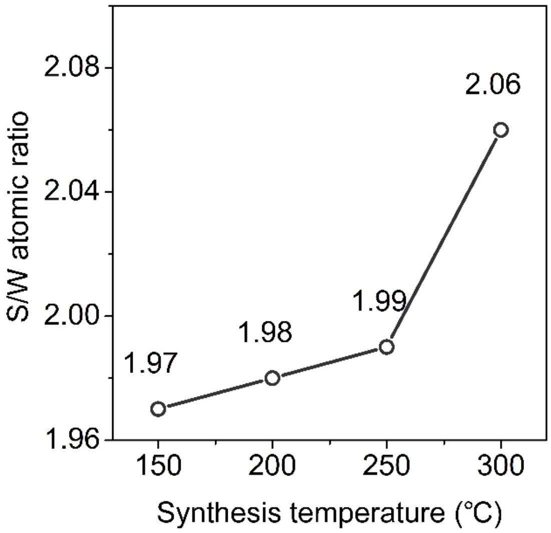

Figure S8. Temperature dependence of the S/W atomic ratio variations of the MP-WS_2 thin film.

Supplementary information 1. Plasma-assisted synthesis process.

A study of the phase transition mechanism in view of both 1) plasma physics and 2) thermodynamics is essential to determine the role of the temperature and plasma properties in the phase modulation during the plasma-assisted MP-WS_2 synthesis processes. Therefore, before the discussion to explain the mechanism, it is essential to review plasma physics [1]. Figure S9 shows the illustration to help understand the plasma-based process. Under an initial plasma, numerous free electrons driven with much higher mobility than ions collide with the exposed W metal thin film, resulting in densely concentrated electrons on the W metal layer (Figure S9a). Subsequently, positive ions, such as Ar^+, can impact the negatively charged W metal layer because of the formed electrical field between the plasma and the metal layer (Figure S9b). At that time, the Ar^+ ions and ions dissociated from H_2S molecules to the metal layer accompany physical and chemical interactions with W atoms as follows (Figure S9c): 1) Inactive Ar^+ ions bombard the W metal layer leaving a defect-fully surface. 2) Sulfur ions permeated through the metal layer are combined with W atoms by forming WS_2 layers. The negative electron flux into the exposed surface decreases until the positive ion flux is equal and repelling to maintain ambipolar flow. The plasma-surface interaction finally reaches a steady state accompanied by the formation of a sheath region near the surface where positive ion flux is the same as the electron flux.

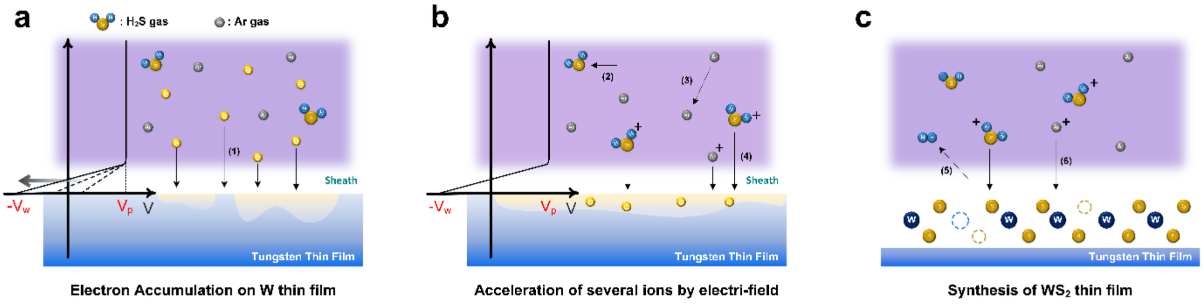

Figure S9. Illustration of the plasma-assisted sulfidation process. a) Electrons accumulation on the W metal thin film. b) The electrical field accelerates positive ions onto the W metal layer. The left graph indicates electrical potential. c) Synthesis of MP-WS_2 thin film: (1) electrons collided with the W metal thin film, (2) penning effect between Ar molecular and H_2S molecular, (3) Ionization, (4) Reaction of positive ions with the W atoms, (5) chemical reaction of sulfur ion, and (6) physical reaction of Ar^+ ion making defects.

To demonstrate the mechanism of the plasma-assisted sulfidation process on the W metal layer, the steel-based Faraday cage was used to examine the effect of positive ions from Ar and H_2S gas molecules (Figure S10a). A Faraday cage with an aperture size of ≈ 40 µm and a height of 3 cm was installed to hinder the electromagnetic field, which generates a plasma. As a result, the W metal layer treated with the cage does not turn completely into MP-WS_2, which consists of four or five layers despite the same environment (Figure S10b). The cage blocks free electrons and ions from reaching the surface.[2] Furthermore, the electrons in the chamber are not sufficiently charged to the W metal layer without the electromagnetic field, reducing the ion bombardment and chemical reaction at the interface. Therefore, in the plasma-assisted sulfidation process, the active species for synthesizing MP-WS_2 are not radical or dissociated atoms but Ar^+ ion bombardment and several sulfur ions. These results support the suggested mechanism.

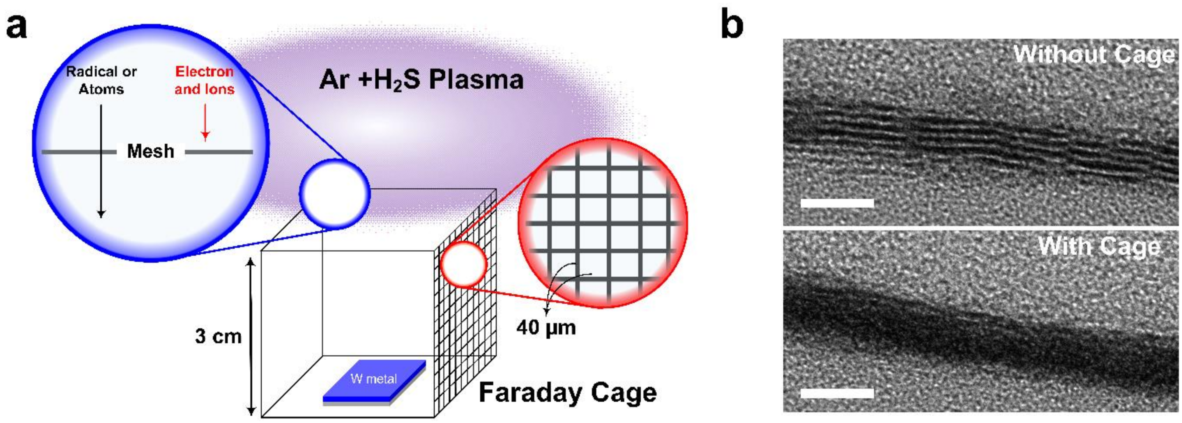

Figure S10. Plasma-assisted sulfidation process with Faraday cage. a) Illustration of the installed Faraday cage during Ar + H_2S plasma. Faraday cage hinders the electromagnetic field and prevents injecting free electrons and ions from the plasma. Radicals or dissociated ions only pass through the mesh. b) Cross-sectional TEM images of the as-synthesized MP-WS_2 depending on the Faraday cage. The scale bar is 5 nm.

Supplementary information 2. Discussion of the structural properties of MP-WS_2.

Based on these HR-TEM results and XRD analysis, it is expected that significant interior strain comes about from the chaotically topological configuration accompanying many defects and grain boundaries (Figure S11). In other words, interfacial strain occurs during simultaneous grain growth from adjacently located nucleation points. Two types of stress can be considered: i) in-plane lattice distortion and ii) out-of-plane expansion.

Out-of-plane direction expansion, as a major contributor to generating interior strain, can be explained with a cross-section view HR-TEM image of the WS_2 layer (Figure S11). The W metal layer of a 1–2 nm thickness is reconstituted to the four or five-layered MP-WS_2 structure with an interlayer spacing of 0.65 nm during the proposed sulfidation process (Figure S11a-b). The atomic force microscopy (AFM) line scanning also displays that the as-prepared WS_2 thin film has a thickness of approximately 3.5 nm in accordance with the TEM measurement (Figure S11c). Moreover, in-plane expansion was confirmed because the WS_2 layers overlapped and bent each other (Figure S11b). Consequently, a series of expansion processes can stimulate the lattice transition, similar to previous studies that have reported a substantial contribution of interfacial strain in TMDC material phase transition.

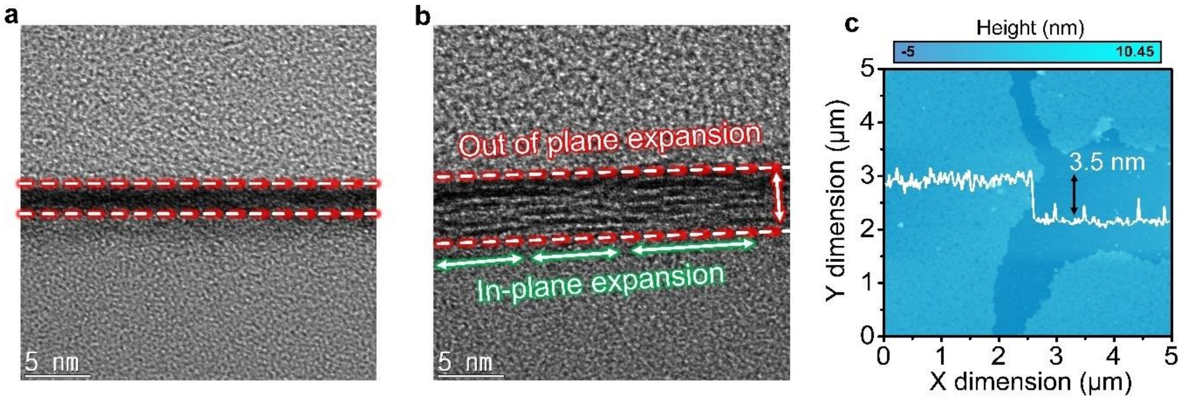

Figure S11. Cross-sectional TEM images of a) W metal layer and b) as-synthesized MP-WS_2 thin film (synthesis time: 1 hour, temperature: 150 °C). c) AFM mapping result of the MP-WS_2 layer, which includes a line profile in the middle of the image.

Supplementary information 3. Overall mechanism of plasma-assisted MP-WS_2 synthesis.

The thermodynamic interaction under an electron-charged environment is considered a major determinant in the phase modulation of the WS_2 layer, not plasma characteristics, according to plasma diagnosis analysis. Persistent electron charging in the plasma environment energetically promotes the transformation of the 1T phase from the 2T phase[3, 4]. Furthermore, the interfacial strains around all sides lead to a phase transition, and the local strain relaxation triggers the increased portion of 2H WS_2 as the process temperature is increased above 150 °C. The complicated synthesis process using plasma suggests the formation mechanism of MP-WS_2 and emphasizes the role of temperature and lattice modification on the phase transition.

Supplementary information 4. Structural and electronic properties using DFT calculation.

To simplify the DFT calculation, it was assumed that there were four cases of vacancies on 1T or 2H WS_2 monolayer: i) one S (V_s), ii) one W (V_w), iii) one S and one W ($V_{s,w}$), and iv) two S and one W ($V_{2s,w}$) are removed. These expressions were followed by the Kröger–Vink notation. The DFT structural relaxation is generally difficult to determine the reliable crystal structures because of its ground state system. The calculated lattice distortion might be different from the TEM analysis above. On the other hand, it has been used to predict the structure and electronic properties of nanomaterials using their final structures[5, 6]. Figure S12 and Table S3-4 provide the geometric information of the initial and relaxed structures.

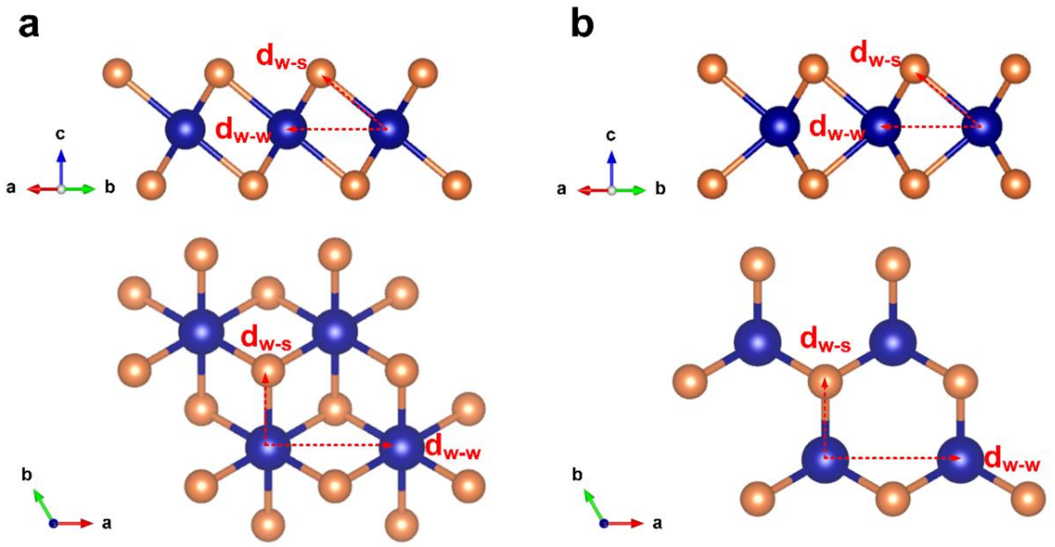

Figure S12. Atomistic configuration of (a) 1T WS_2 and (b) 2H WS_2. (c) Information of modified d_{s-w} and d_{w-w} for (Top) pristine 1T WS_2 and 2H WS_2 and (Bottom) relaxed 1T WS_2 and 2H WS_2 with several vacancies.

Table S2. Atomic configuration presented in Figure S11. The atomic distances of the initial structures for pristine 1T WS_2 and 2H WS_2.

Before	1T WS_2				2H WS_2			
Distance [Å]	V_s	V_w	$V_{s,w}$	$V_{2s,w}$	V_s	V_w	$V_{s,w}$	$V_{2s,w}$
d_{s-w}	2.43	2.43	2.43	2.43	2.42	2.42	2.42	2.42
d_{w-w}	3.25	3.25	3.25	3.25	3.25	3.25	3.25	3.25

Table S3. Atomic configuration presented in Figure S11. Atomic distances of the relaxed structures for 1T WS_2 and 2H WS_2 with vacancies. The table is filled in as the minimum and maximum distances modified by structural distortion.

Relaxation	1T WS_2				2H WS_2			
Distance [Å]	V_s	V_w	$V_{s,w}$	$V_{2s,w}$	V_s	V_w	$V_{s,w}$	$V_{2s,w}$
d_{s-w}	2.34/2.48	2.32/2.44	2.32/2.46	2.23/2.55	2.41/2.42	2.38/2.43	2.35/2.46	2.35/2.43
d_{w-w}	2.99/3.53	2.85/3.13	2.75/3.79	2.81/3.30	3.15/3.19	3.12/3.21	2.82/3.32	2.78/3.41

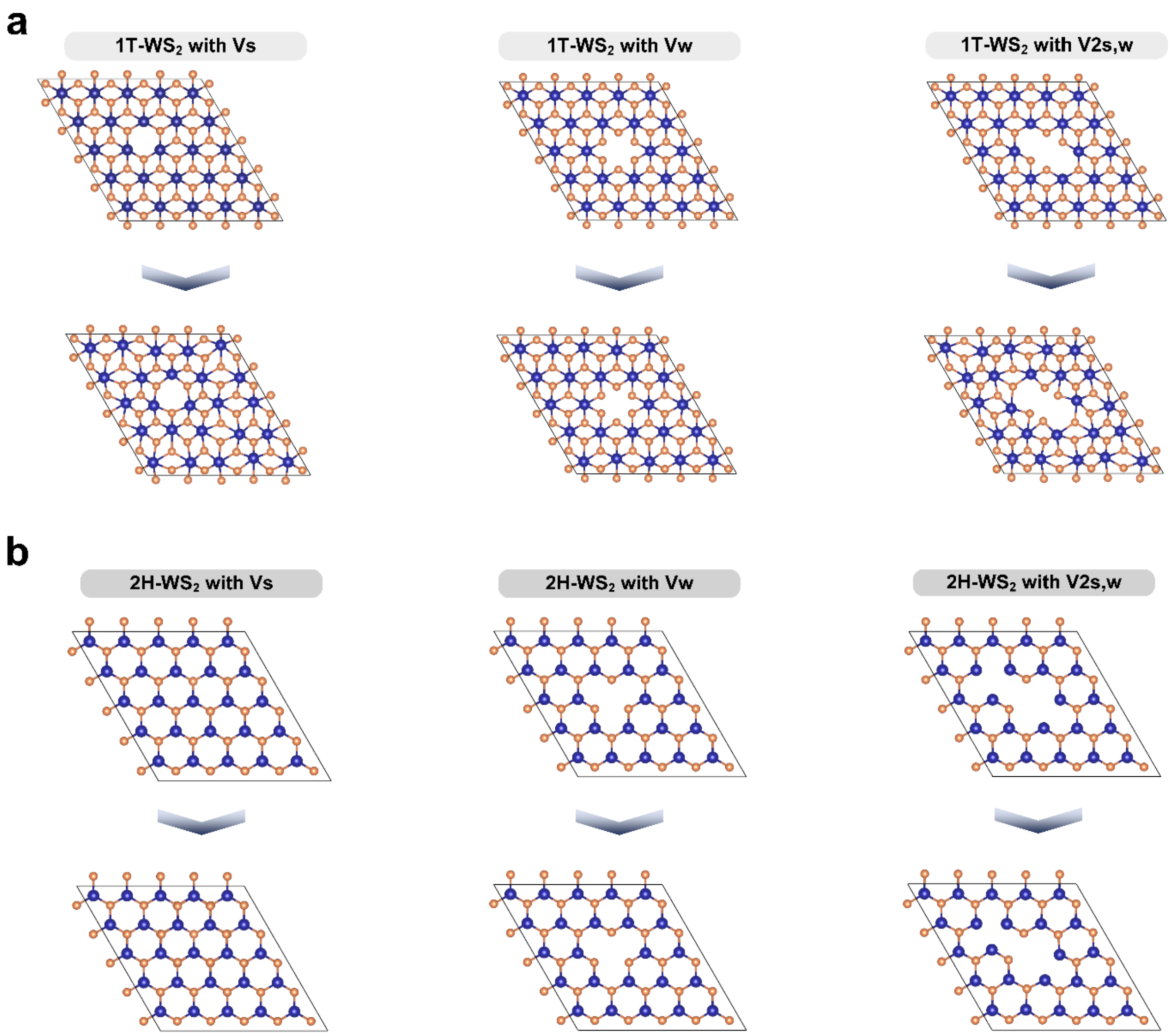

Figure S13. Atomistic configuration of 1T WS_2 with (a) V_s, (b) V_w, (c) $V_{w,s}$, and (d) $V_{w,2s}$, and 2H WS_2 with (e) V_s, (f) V_w, (g) $V_{w,s}$, and (h) $V_{w,2s}$. Each figure exhibits the atomistic configuration after structural relaxation.

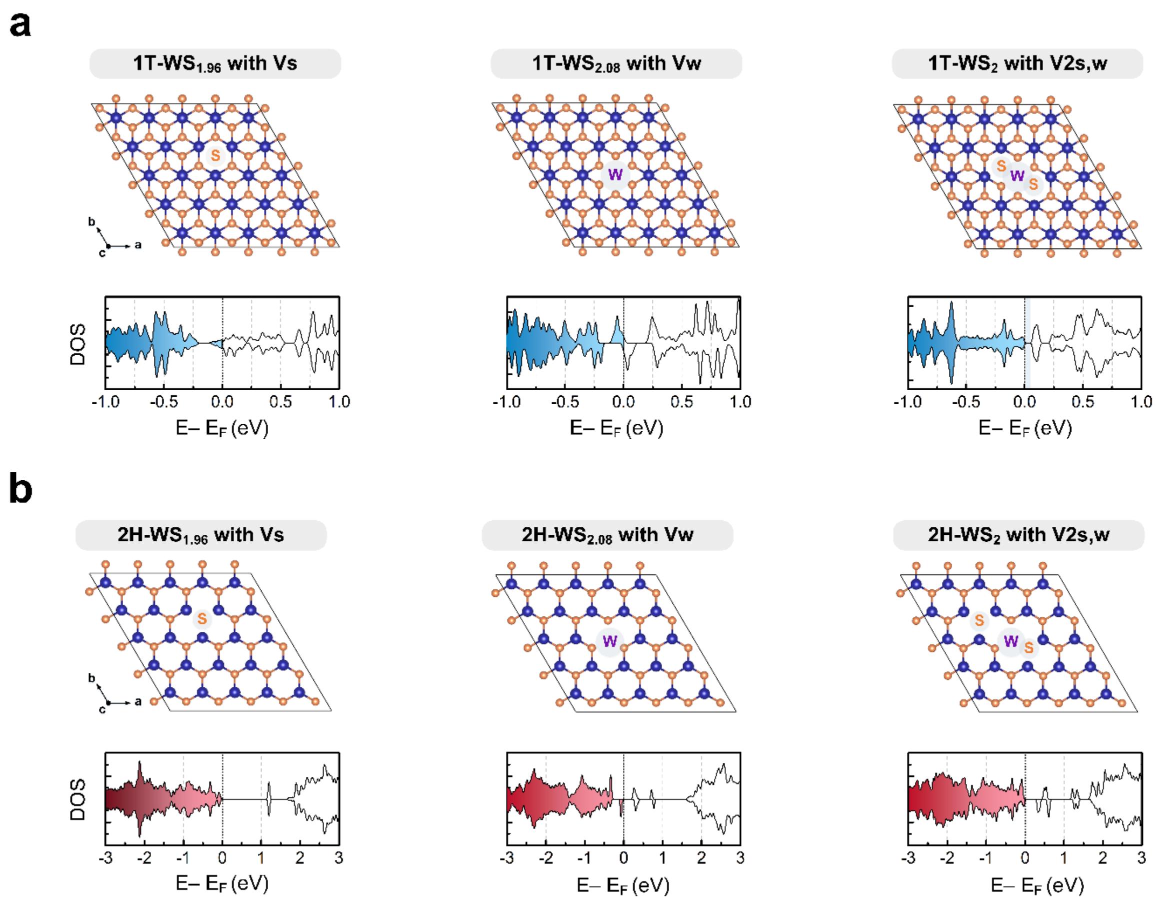

Figure S14. Total density of states (TDOS) of (a) 1T WS_2 and (b) 2H WS_2 depending on several vacancies (V_s, V_w, and $V_{w,2s}$).

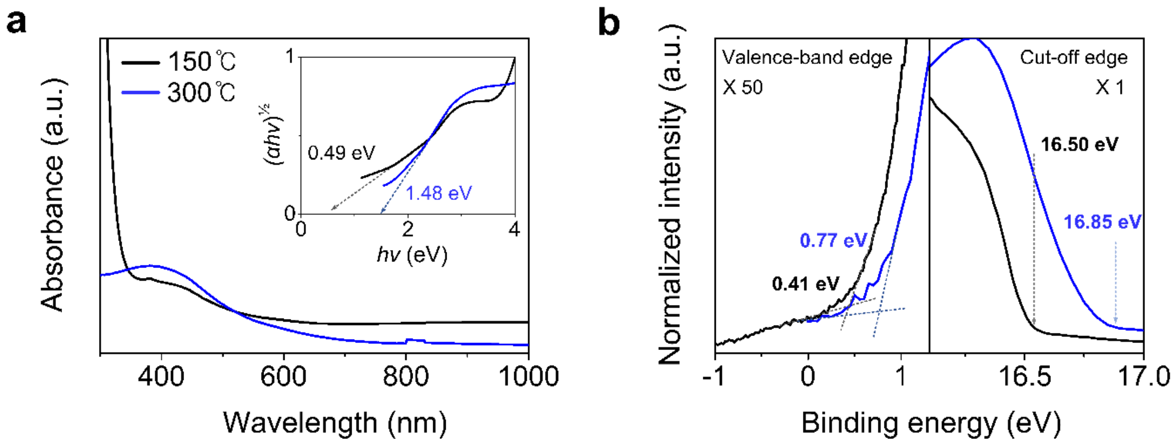

Figure S15. Electronic structure of MP-WS_2. (a) UV-vis and (b) UPS results for the MP-WS_2 thin film synthesized at 150 °C and 300 °C.

Table S4. Band characteristic of MP-WS_2 prepared at 150 and 300 ℃ and *p*-Si.

**Materials**	**Valence edge (eV)**	**Cutoff edge (eV)**	**Fermi level (eV)**	**Ionization energy (eV)**	**Electron Affinity (eV)**	**Bandgap (eV)**
p-Si[7]	-	-	5.00	5.21	4.08	1.13
MP-WS_2 150 ℃	0.41	16.5	4.72	5.13	4.64	0.49
MP-WS_2 300 ℃	0.77	16.85	4.37	5.14	3.66	1.48

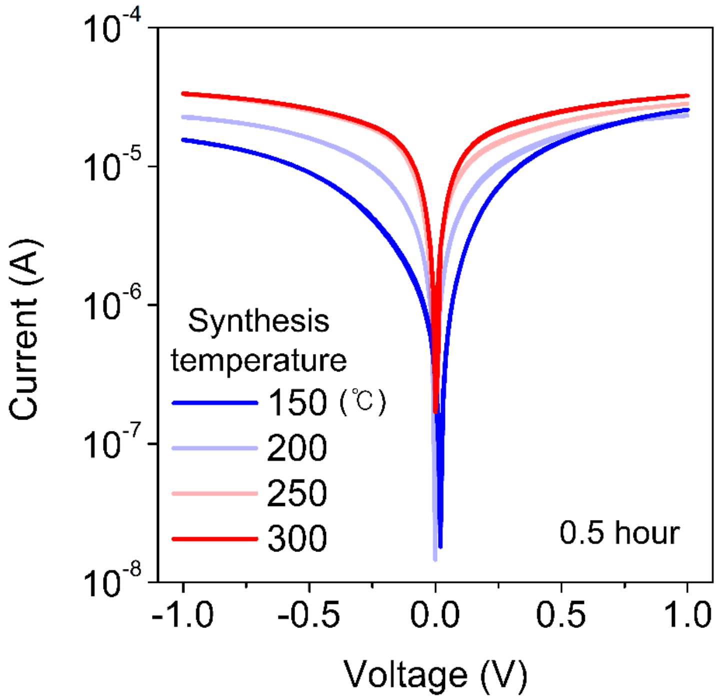

Figure S16. *I*-*V* curve of the MP-WS_2/*p*-Si heterostructure prepared at 0.5 hours and 150 to 300 ℃

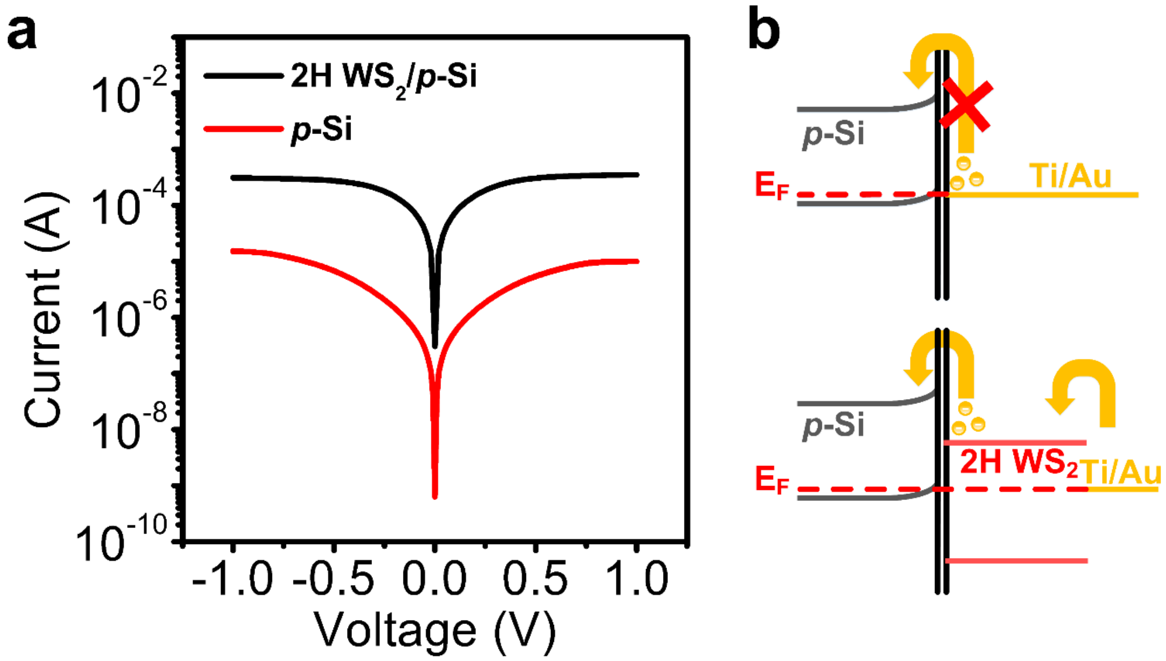

Figure S17. (a) Comparison of the photocurrent of 2H WS_2/*p*-Si heterostructure with *p*-Si photodiode under halogen light irradiation with 5.3 mW/cm^2. (b) Band diagram of 2H Au/Ti/WS_2/*p*-Si heterostructure and Au/Ti/*p*-Si.

Supplementary information 5. Discussion on the NDR operation mechanism

Although MP-WS_2 is not fully isolated on the *p*-Si substrate in each single device, the major carrier transportation channel is formed through the vertical direction rather than the lateral direction (Figure S18b). Nano-grained 2D materials do not exhibit good conductance. On the other hand, the *p*-Si substrate shows good crystallinity. Hence, the total current behavior mainly reflects the MP-WS_2/*p*-Si junction effect. The process for NDR generation can be explained through a three-step procedure and applied V_D variations (Figure S18). Initially, the MP-WS_2/*p*-Si heterostructure has symmetric band alignment when the V_D between the source and drain electrode is zero (Figure S18c). When the positive bias is applied on the electrode, the NDR behavior of the MP-WS_2/*p*-Si device is derived from the heterojunction below the source electrode (yellow box)(Figure R18d-e) i) At $V_D < 0.3$ V, photodoping effect emerged by photo-excited electrons, which accumulated at the interface between *p*-Si and D-1T WS_2, enlarging the tunneling window and promoting electron tunneling. Throughout the broken gap, the current varies linearly with increasing V_D (Figure S18e). ii) At 0.3 V < V_D < 0.5 V, the conduction band of D-1T WS_2 is aligned with the forbidden gap of *p*-Si, blocking electron tunneling and forming a current-decreasing region. iii) When V_D exceeds 0.5 V, the contribution of thermionic current to the total current increases as electrons jump up to the conduction band of *p*-Si beyond the built-in potential. This increases the total current and presents 'N'-shaped current behavior, as shown in Figure S18d. Reversely, the main driver of NDR behavior changes to the heterojunction below the drain electrode according to applying negative bias (orange box). The NDR behavior operation along to apply negative bias is same with the case of applying positive bias. Electron tunneling (-0.3 V < V_D < 0 V), transition of electron transportation mechanism (-0.5 V < V_D < -0.3 V), and diffusion current (V_D < -0.7 V) is consecutively exhibited, resulting in clear NDR behavior at negative bias side. Consequently, a symmetric NDR curve is generated (Figure R18f-g).

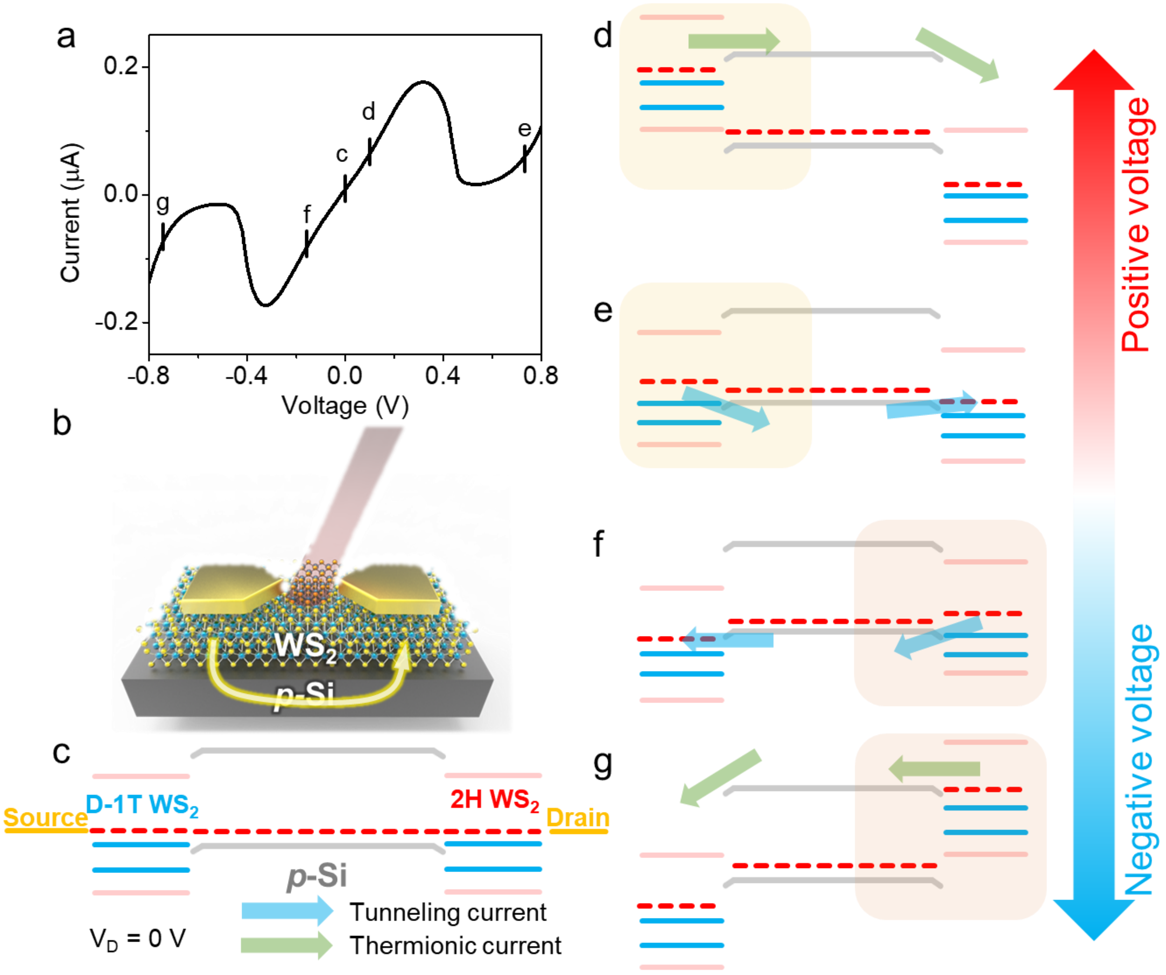

Figure S18. Operation mechanism of the photo-reactive NDR device. a) Linear-scale *I*-*V* curve of the proposed NDR device. b) Schematic of the WS_2/*p*-Si heterostructure. band alignment of the proposed NDR device at the drain voltage of c) 0 V, d) 0.7 V, e) 0.1 V, f) -0.15 V, and g) -0.7 V, respectively.

Table S5. Performance comparison of the proposed MP-WS_2/*p*-Si heterostructure with previously reported NDR devices.

Materials	Peak current (μA)	PVCR$^{\alpha}$ (A/A)	Modification method	Uniformity test (#)	Year	Ref
HfS_2/Pentacene	3.20×10^{-12}	2.2	Intrinsic materials properties	4	2020	Ref[8]
MoS_2/BP	9.00×10^{-10}	1.22	Gating and hBN encapsulation	3	2021	Ref[9]
Bi_2WO_6	5.50×10^{-11}	N/A$^{\gamma}$	Ferroelectric material properties	N/A	2022	Ref[10]
b-As/$SnS_2$$^{\beta}$	3.00×10^{-7}	4.6	Intrinsic materials properties	3	2022	Ref[11]
MoS_2	3.00×10^{-11}	1.5	Defect engineering	N/A	2022	Ref[12]
$MoTe_2$/HAT-CN	1.60×10^{-10}	137	Intrinsic materials properties	N/A	2020	Ref[13]
V-doped WSe_2/$SnSe_2$	2.40×10^{-6}	1.6	Chemical doping	N/A	2020	Ref[14]
MP-WS_2/*p*-Si	2.02×10^{-7}	13.8	Phase modulation	140	This work	

$^{\alpha}$ Peak-to-valley ratio
$^{\beta}$ Black arsenic
$^{\gamma}$ Not available

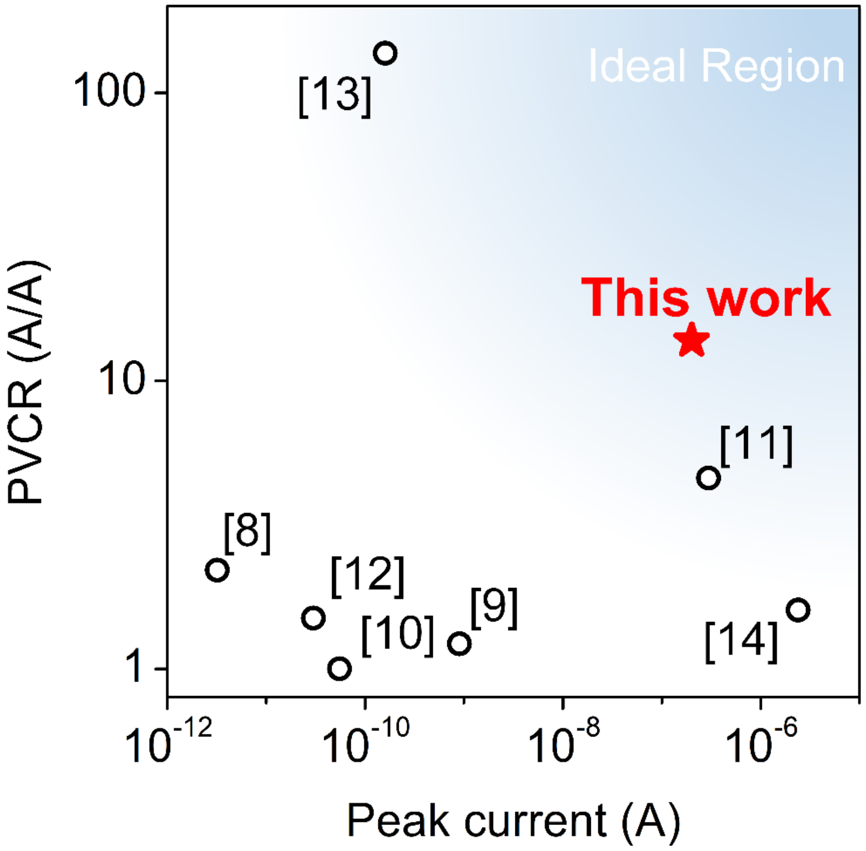

Figure S19. PVCR vs. Peak current plot of the proposed device performance and previously reported NDR devices.

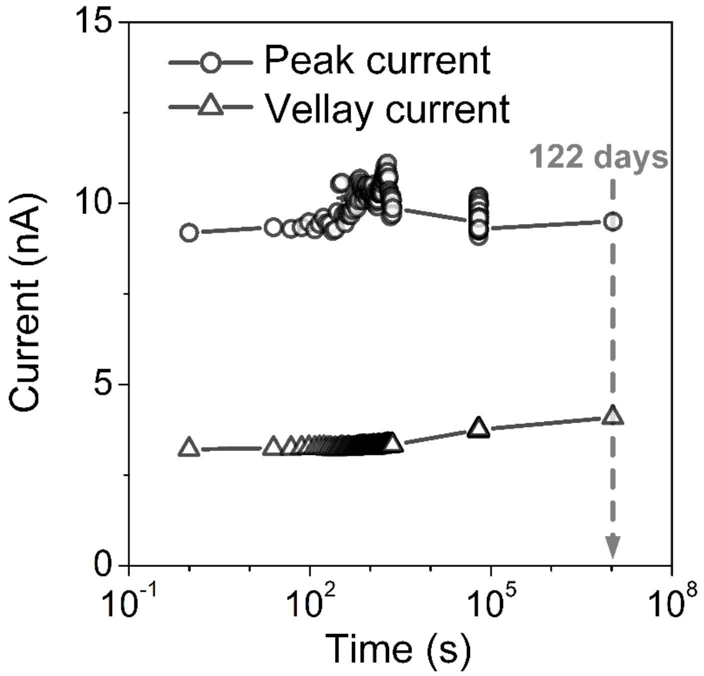

Figure S20. Long-term stability test of the MP-WS_2/*p*-Si heterostructure under illumination.

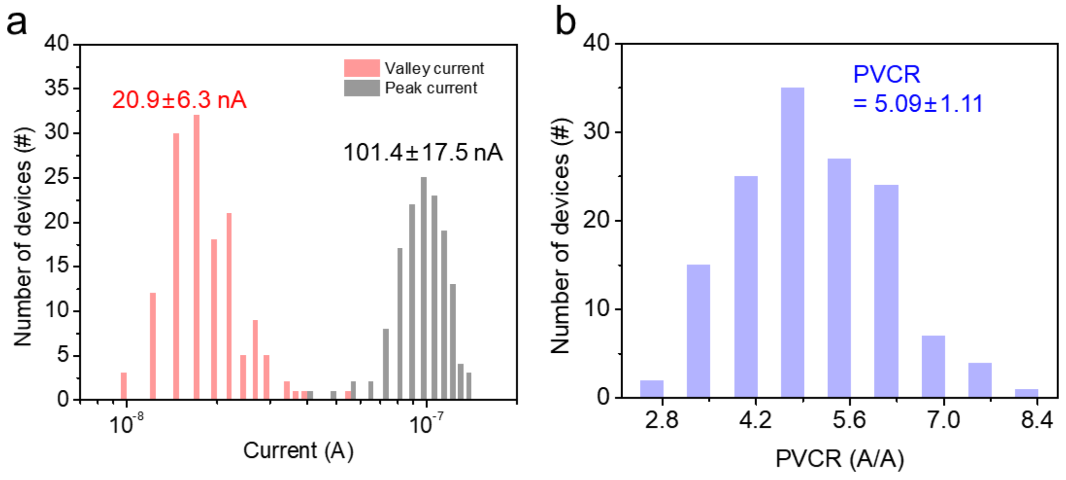

Figure S 21. a) Current and b) PVCR distribution of the 140 proposed NDR devices

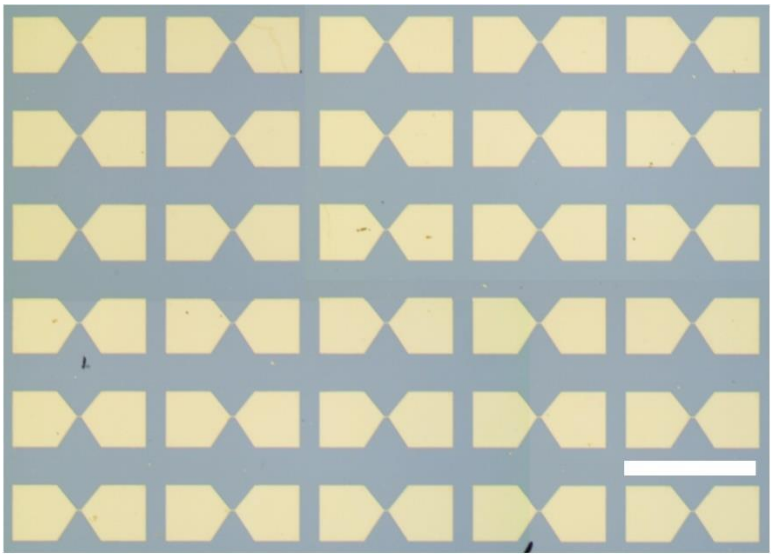

Figure S22. Image of a 5 × 6 NDR device array with a channel length of 3 μm (scale bar = 500 μm)

Supporting References

[1] T. Panagopoulos, D. J. Economou, *J. Appl. Phys.* **1999**, 85, 3435-3443.

[2] Y. Qi, B. Demg, X. Guo, S. L. Chen, J. Gao, T. R. Li, Z. P. Dou, H. N. Ci, J. Y. Sun, Z. L. Chen, R. Y. Wang, L. Z. Cui, X. D. Chen, K. Chen, H. H. Wang, S. Wang, P. Gao, M. H. Rummeli, H. L. Peng, Y. F. Zhang, Z. F. Liu, *Adv. Mater. 2018*, 30, 1704839.

[3] X. M. Yin, Q. X. Wang, L. Cao, C. S. Tang, X. Luo, Y. J. Zheng, L. M. Wong, S. J. Wang, S. Y. Quek, W. J. Zhang, A. Rusydi, A. T. S. Wee, *Nat. Commun.* **2017**, 8, 486.

[4] G. P. Gao, Y. Jiao, F. X. Ma, Y. L. Jiao, E. Waclawik, A. J. Du, *J. Phys. Chem. C* **2015**, 119, 13124-13128.

[5] K. Frohna, T. Deshpande, J. Harter, W. Peng, B. A. Barker, J. B. Neaton, S. G. Louie, O. M. Bakr, D. Hsieh, M. Bernardi, *Nat. Commun.* **2018**, 9, 1829.

[6] Y. L. Yue, X. Y. Zhang, J. Q. Qin, R. P. Liu, *J. Alloys Compd.* **2021**, 860, 157906.

[7] S. Kment, F. Riboni, S. Pausova, L. Wang, L. Wang, H. Han, Z. Hubicka, J. Krysa, P. Schmuki, R. Zboril, *Chem. Soc. Rev.* **2017**, 46, 3716-3769.

[8] K. S. Jung, K. Heo, M. J. Kim, M. Andreev, S. Seo, J. O. Kim, J. H. Lim, K. H. Kim, S. Kim, K. S. Kim, G. Y. Yeom, J. H. Cho, J. H. Park, *Adv. Sci.* **2020**, 7, 2000991.

[9] F. Wu, H. Tian, Z. Y. Yan, J. Ren, T. Hirtz, G. Y. Gou, Y. Shen, Y. Yang, T. L. Ren, *ACS Appl. Mater. Interfaces* **2021**, 13, 26161-26169.

[10] C. Y. Song, H. C. Mao, Y. B. Yang, X. Liu, Z. P. Yin, Z. P. Hu, K. H. Wu, J. X. Zhang, *Adv. Funct. Mater*. **2022**, 32, 2105256.

[11] G. Dastgeer, S. Nisar, Z. M. Shahzad, A. Rasheed, D. K. Kim, S. H. A. Jaffery, L. Wang, M. Usman, J. Eom, *Adv. Sci.* **2023**, 10, 2204779.

[12] W. H. Chang, C. I. Lu, T. H. Yang, S. T. Yang, K. B. Simbulan, C. P. Lin, S. H. Hsieh, J. H. Chen, K. S. Li, C. H. Chen, T. H. H1ou, T. H. Lu, Y. W. Lan, *Nanoscale Horiz.* **2022**, 7, 1533-1539.

[13] W. Choi, S. Hong, Y. Jeong, Y. Cho, H. G. Shin, J. H. Park, Y. Yi, S. Im, *Adv. Funct. Mater.* **2021**, 31, 2009436.

[14] S. Fan, S. J. Yun, W. J. Yu, Y. H. Lee, *Adv. Sci.* **2020**, 7, 1902751.
